# A chronopharmacological comparison of ciprofol and propofol: focus on sedation and side effects

**DOI:** 10.3389/fnmol.2025.1567453

**Published:** 2025-04-02

**Authors:** Xuehan Li, Xinqing Yang, Chen Chen, Ziqing Yu, Houping Wang, Ruixue Liu, Jianrong He, Bin Shu, Guangyou Duan, Erquan Zhang, Dapeng Ju, He Huang

**Affiliations:** ^1^Department of Anesthesiology, The Second Affiliated Hospital, Chongqing Medical University, Chongqing, China; ^2^National Institute of Biological Sciences, Beijing, China

**Keywords:** chronopharmacology, ciprofol, critical anesthesia dosage, drug distribution, GABAAR, propofol

## Abstract

Propofol is a commonly used intravenous anesthetic in clinical practice, while ciprofol, a propofol derivative, also targets GABAA receptors with enhanced anesthetic potency. Regarding chronopharmacology, it remains unclear whether the new drug ciprofol has improved anesthetic effect and less side effects compared with propofol. First, we assessed the critical anesthetic dosage (D_ca_) of ciprofol and propofol exhibited diurnal rhythmicity. At the highest D_ca_, the loss of righting reflex duration was significantly longer for ciprofol than that for propofol at both Zeitgeber Time (ZT) 4 and ZT16. The β_3_ subunits of the GABAA receptor, which are involved in mediating anesthetic effects, and the metabolizing enzyme UGT1A9 for propofol demonstrated rhythmic expression. Moreover, molecular dynamics simulation indicated a higher binding affinity of R-ciprofol to GABRB3 compared with propofol. Animal behavior experiments indicated that ciprofol was associated with no incidence of side effects at any time of day, while propofol exhibited circadian-related adverse effects. Notably, ciprofol infrequently disrupted the rhythmicity of clock gene expression compared to propofol. From a chronopharmacological perspective, ciprofol offers improved sedation and fewer side effects compared to propofol, suggesting its higher potential for clinical application.

## Graphical abstract

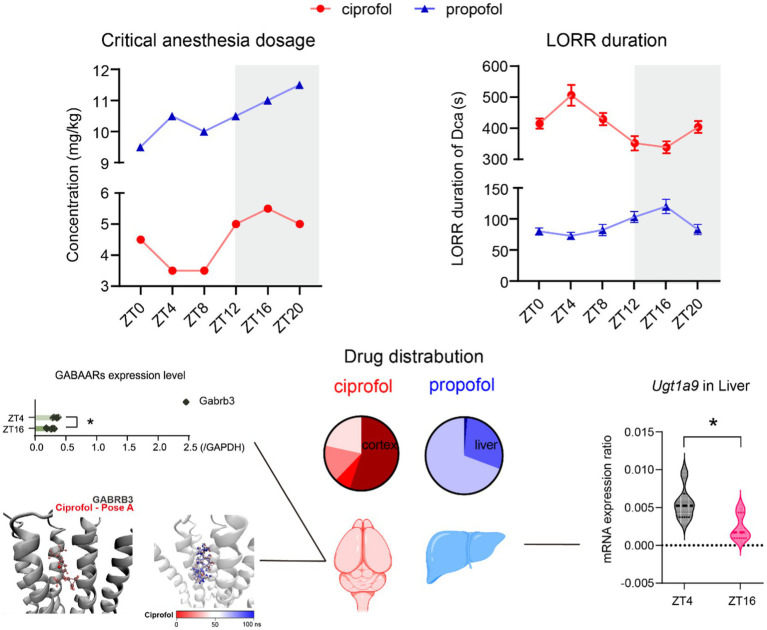

## Highlights

- Both ciprofol and propofol exhibit diurnal variations in anesthetic effective concentration.
- The diurnal rhythm of propofol may be driven by its metabolism, whereas that of ciprofol appears to be mediated by central receptors expression pattern.
- The side effects of propofol vary between day and night, whereas ciprofol appears to have no significant side effects.

## 1 Introduction

The circadian rhythm regulates the 24-h diurnal variations in numerous aspects of human physiology and drug responses (Panda et al., 2002). Chronopharmacology is a discipline that examines the influence of circadian rhythms on the pharmacokinetics and pharmacodynamics of medications (Fujimura and Ushijima, 2023), aiming to optimize the timing of drug administration for maximum therapeutic efficacy while minimizing dosages and side effects (Chowdhury et al., 2019). Anesthetics, typically used for short-term interventions, show variability in their effects throughout the day and night (Sugano et al., 2021; Kilicarslan et al., 2021). Moreover, these anesthetic agents are associated with significant side effects, including delirium, cognitive dysfunction, and memory impairment (Tang et al., 2023; Ni et al., 2024). Furthermore, the non-anesthetic effects of these agents may persist for several days or even weeks, potentially prolonging hospital stay and diminishing patients’ quality of life (Song et al., 2021). Therefore, it is crucial to investigate the chronopharmacology of anesthetic drugs to enhance patient outcomes and reduce adverse effects.

Propofol is a widely used intravenous anesthetic known for its rapid onset and quick elimination. Ciprofol, a derivative of propofol, also targets GABAA receptors to induce anesthetic effects (Qin et al., 2017). The incorporation of cyclopropyl group(s) in ciprofol enhances its lipophilicity, resulting in a low effective dosage and fast distribution in the brain. Although it has been reported that circadian rhythms can influence the depth of anesthesia achieved with propofol (Shen et al., 2021), it remains unclear whether the effective concentrations of propofol and ciprofol vary throughout the day and whether their circadian rhythmicity is affected. Therefore, a comprehensive investigation into the diurnal profile of the sedative effects of ciprofol compared to those of propofol could facilitate adjustments to dosage regimens based on diurnal patterns, allowing for a more precise anesthesia administration.

While propofol is effective in inducing anesthesia, it is also associated with serious side effects, including delirium, cognitive dysfunction, and memory impairment, as well as circadian rhythm disruptions (Challet et al., 2007). These adverse effects can be particularly severe and prolonged in high-risk or hypersensitive patients (Li et al., 2024). It remains uncertain whether ciprofol, due to its structural modifications, has inherited similar side effects to those observed with propofol. A comprehensive evaluation from a chronopharmacological perspective is necessary to determine whether these side effects exhibit diurnal characteristics and how they may differ from those associated with propofol.

To fully understand the circadian features of propofol and ciprofol regarding their sedation and side effects, we conducted a study to explore the critical anesthesia dosage (D_ca_) and sedation effect of both agents throughout the day and further evaluated the side effects of propofol and ciprofol during the light and dark phases.

## 2 Materials and methods

### 2.1 Animals

Male C57/BL6J mice (8–10-week-old) were maintained in a pathogen-free environment at a constant temperature of 23°C (22–24°C) under a 12-h light/12-h dark cycle. All animals had ad libitum access to food and water. All procedures were approved by the Institutional Animal Care and Use Committee of the National Institute of Biological Sciences, Beijing, in accordance with Chinese regulations. The mice were obtained from Vital River and GemPharmatech.

### 2.2 Exploration of D_ca_

Diurnal variations in anesthesia dosage were assessed over a 24-h period at 4-h intervals, starting at ZT0. The D_ca_ (critical anesthetic dosage) at each time point was determined based on the LORR (Loss of Righting Reflex) rates of a group of mice (n = 7). At each time point, three ascending concentration gradients were tested: C1, C2, and C3, with an interval of 0.5 mg/kg between each concentration. Specifically, when the LORR rate at a lower dose was less than 100%, and the LORR rate at the higher dose reached 100%, the higher dose was defined as the D_ca_ for that time point. This method allows us to identify the minimal dose required to achieve a complete LORR effect. Initial C2 dosages for ciprofol (Haisco, 2.5 mg/mL) were 4.5 mg/kg at ZT0, 5 mg/kg at ZT4, 5.5 mg/kg at ZT8, 6 mg/kg at ZT12, 6.5 mg/kg at ZT16, and 5 mg/kg at ZT20. For propofol (Fresenius Kabi, 10 mg/mL), the C2 dosages were 10.5 mg/kg, 11 mg/kg, 11.5 mg/kg, 12 mg/kg, 12.5 mg/kg, and 12 mg/kg, respectively. Only mice that received successful drug injections on the first attempt were included in the analysis. Each concentration test was completed within 15 min.

### 2.3 Exploration of LORR duration

The highest D_ca_ for each drug (ciprofol 5.5 mg/kg, propofol 11.5 mg/kg) was adopted to assess diurnal variations in LORR duration. Measurements were taken at 4-h intervals over a 24-h period. Mice received a single tail vein injection, and the duration of the procedure was kept within 15 min. The LORR durations were recorded.

### 2.4 HPLC to detect drug concentration

Eighty mice were randomly divided into four groups: ZT4 ciprofol (5.5 mg/kg), ZT4 propofol (11.5 mg/kg), ZT16 ciprofol (5.5 mg/kg), and ZT16 propofol (11.5 mg/kg). Each group was further subdivided into four groups, namely 0, 2, 5, and 8 min (n = 3 ~ 5) after tail vein administration. Blood samples were immediately collected after euthanasia (Tribromoethanol, 240 mg/kg, I.P., 0.25%), along with tissues, such as the hypothalamus, cortex, and liver, which were quickly placed in liquid nitrogen. Blood samples were centrifuged at 5000 rpm for 15 min at 4°C, and the supernatant was transferred to a new 1.5 mL EP tube and also stored in liquid nitrogen before being frozen at −80°C.

For analysis, 50 μL of plasma was carefully aspirated and centrifuged to obtain the vortexed and centrifuged supernatant. The resulting supernatant was then transferred into a clean sample bottle for LC–MS/MS analysis using blank plasma samples to form a standard curve. We weighed 0.1 g of each sample and rapidly cooled them using liquid nitrogen. The tissue samples were homogenized using a RETSCH MM400 grinder (Germany), then vortexed and subjected to low-temperature ultrasound extraction, followed by centrifugation at 4°C. The supernatant was collected, dried, and reconstituted with 100 μL of 50% methanol. The mixture was vortexed for 5 min and subjected to ultrasound for 10 min, followed by centrifugation at 13,000 rpm at 4°C for an additional 10 min to obtain the supernatant for LC–MS/MS analysis. Concurrently, a standard curve was created using blank tissue samples.

### 2.5 Western blot analysis of protein levels

At two time points, ZT4 and ZT16, whole brains from the mice were collected after euthanasia (Tribromoethanol, 240 mg/kg, I.P., 0.25%) (*n* = 6). The samples were mechanically homogenized by drill-driven pestles and sonicated 10 times for 10 s each. The homogenate was heated at 70°C for 5 min and centrifuged at 21,000 g for 10 min at 4°C. The supernatant was isolated, and the total protein concentration was determined using a microvolume spectrophotometer (Nanodrop, Thermo Fisher). The supernatant was subjected to drop electrophoresis, transferred to a membrane, blocked, and incubated with primary and secondary antibodies. Protein levels were visualized using an ODYSSEY CLX exposure machine, and gray values were analyzed with Image J-Fiji software. The antibody source are listed in Table 1. The uncropped original western blots was in Supplementary material WB data s, and the original uncropped original western blots with pvdf membraine and film were in Supplementary material WB pvdf and film.

**Table 1:** Antibodies used in Western blot analysis.

Antibodies	Source	Identifier
Anti-GABA(A)α1Receptor	Alomone Labs	#AGA-001
Anti-GABA(A)α2Receptor	Alomone Labs	#AGA-002
Anti-GABA(A)α3Receptor	Alomone Labs	#AGA-003
Anti-GABA(A)α4Receptor	Alomone Labs	#AGA-004
Anti-GABA(A)α5Receptor	Alomone Labs	#AGA-005
Anti-GABA(A)α6Receptor	Alomone Labs	#AGA-006
Anti-GABA(A)β3Receptor	ABclonal	A10015
Anti-GABA(A)γ2Receptor	ABclonal	A1733
GAPDH Mouse mAb (High Dilution)	ABclonal	Cat# AC033
Anti-rabbit HRP-linked secondary	Cell Signaling Technology	Cat# 7074; RRID:AB_2099233
Anti-mouse HRP-linked secondary	Cell Signaling Technology	Cat# 7076; RRID:AB_330924

### 2.6 Molecular dynamics simulation

The CryoEM structures of GABRB3 (PDB ID: 7A5V) and propofol with GABA receptor α_1_β_2_γ_2_ subtype (PDB ID: 6X3T) were used for model construction. α_1_β_2_γ_2_ pentamer in 6X3T was aligned with GABRB3 pentamer in 7A5V, and the coordinate of propofol in 6X3T was copied to GABRB3 directly. Both poses of R-ciprofol was derived from propofol with Gaussian (Gaussian 16 Rev, 2016). The topology of propofol and ciprofol were built with acpype (Sousa da Silva and Vranken, 2012) using GAFF force field (Wang et al., 2006; Wang et al., 2004) and AM1-BCC charge. Molecular dynamics simulation was performed to relax the complex with GROMACS 2023.1 (Berendsen et al., 1995). Briefly, the simulation used amber14sb_parmbsc1 forcefield for protein (Yu et al., 2024). The ciprofol/propofol-GABRB3 complex was placed in a triclinic box with periodic boundary, which was filled with TIP3P water molecules and was neutralized with 150 mM NaCl solution. The system was energy minimized until the maximum force on any atom was less than 100 kJ/mol-1 nm-1, following equilibrated for 200 ps. A 100 ns molecular dynamics (MD) simulation was then completed. V-rescale thermostat was applied with 298.15 K and 0.2 ps tau-t. The complex was set in one temperature controlling group, and the environment set in another group. MMPBSA calculations was carried by gmx_MMPBSA program with 4.0 fillratio and 0.15 istrng.

### 2.7 Open field test

To assess spontaneous locomotor activity and anxiety-like behavior in the animals, The open field test was conducted in ZT4 and ZT16. The apparatus consisted of a square open field arena (50 cm × 50 cm × 37 cm). The arena was placed in a quiet, dimly lit room to minimize external disturbances. Before the test, animals were acclimated to the experimental room for at least 1 h to reduce the effects of environmental stress. The open field arena was thoroughly cleaned with 75% ethanol and dried before each trial to remove any olfactory cues from previous animals. Each animal was gently placed in the center of the open field arena and allowed to explore freely for a period of 8 min. The behavior of the animals was recorded using a digital camera mounted above the arena, and the data were analyzed using automated video-tracking software (SMART 3.0). The following parameters were measured and analyzed: The total distance, mean speed, time spent in the center/peripheral zone (central zone: the central 1 square).

### 2.8 New and old object recognition experiment test

To assess short-term (2-h) cognitive learning function in mice 3 and 24 h after administration, a familiarization period was ensured (object AA) 1 h after administration, followed by a testing phase (object AB) 2 h later in the 3-h group. A second familiarization period (object AA) was ensured 22 h after administration, leading to a second testing phase (object AC) 2 h later in the 24-h group.

### 2.9 Quantitative PCR validation

qPCR was utilized in two distinct sets of experiments. Generally, all mice were under LD (Light: Dark) conditions 1 week before experiment. 1. On the 8th day at ZT4 and ZT16, the whole brain, kidney, and liver were collected from the mice after euthanasia (Tribromoethanol, 240 mg/kg, I.P., 0.25%) (*n* = 6). 2. The mice were randomly divided into six groups (n = 18 per group), with each group evenly allocated across six time points (*n* = 3 per time point): ZT4 saline, ZT4 ciprofol, ZT4 propofol, ZT16 saline, ZT16 ciprofol and ZT16 propofol. On the 8th day, single tail vein injections were administered at ZT4 and ZT16 in each group (Saline: 2.75 mL/kg; Ciprofol: 5.5 mg/kg; Propofol: 11.5 mg/kg). The hypothalamus were collected from the mice after euthanasia (Tribromoethanol, 240 mg/kg, I.P., 0.25%). Notably, the injection procedure at ZT16 was performed under dim light conditions in about 5 min.

Samples were stored at −80° C. After mechanical homogenization by drill-driven pestles in TissueMaster™ (TissueMaster™ High-Throughput Tissue Homogenizer) (60 Hz for 120 s) in trizol and 10 times sonication for 10 s, the brain samples were heated at 70°C for 5 min and centrifuged at 21000 g for 10 min at 4°C. The supernatant was isolated, and the total protein concentration was determined using a microvolume spectrophotometer (Nanodrop, Thermo Fisher).

Quantitative PCR primer sets for *Gabra1-6, Gabrg2, Gabrb3, Ugt1a9* genes and the circadian genes *Nr1d1*, *Per1* and *Dbp* were designed using the National Center for Biotechnology Information primer design. Quantitative analysis of the mRNA volume was performed via real-time PCR using iTaq SYBR Green Supermix from Bio-Rad Laboratories, Inc. (2000 Alfred Nobel Drive, Herles, CFX96 Real-Time System). Gene expression was calculated by comparing with GAPDH. The primer sequences are listed in Table 2.

**Table 2:** Primers used in real-time fluorescence quantitative-PCR.

RNA primer		
GABRA1	Forward primer	AAAAGTCGGGGTCTCTCTGAC
	Reverse Primer	CAGTCGGTCCAAAATTCTTGTGA
GABRA2	Forward Primer	GGACCCAGTCAGGTTGGTG
	Reverse Primer	TCCTGGTCTAAGCCGATTATCAT
GABRA3	Forward Primer	ATGTGGCACTTTTATGTGACCA
	Reverse Primer	CCCCAGGTTCTTGTCGTCTTG
GABRA4	Forward Primer	ACAATGAGACTCACCATAAGTGC
	Reverse Primer	GGCCTTTGGTCCAGGTGTAG
GABRA5	Forward Primer	TGACCCAAACCCTCCTTGTCT
	Reverse Primer	GTGATGTTGTCATTGGTCTCGT
GABRA6	Forward Primer	GTCGGATTCTTGACAACTTGCT
	Reverse Primer	AGATGTCTGTTTTGACTTCTGT
GABRB3	Forward Primer	ACCGTCTGGTCTCCAGGAATG
	Reverse Primer	GATCAGGATTGAGGGCATA
GABRG2	Forward Primer	GCTCTACCCAGGCTTCACAAG
	Reverse Primer	CCAGCAGGTTGTTTAAGATGACA
CYP2B10	Forward Primer	CCCCCATGTTGCAGAGAAAGTC
	Reverse Primer	GCCTTGGAGCCCTGGAGATTT
UGT1A9	Forward Primer	CAATCCCTCAGAGCATCA
	Reverse Primer	GCCACTGTCCCTGTCAAA
NR1D1	Forward Primer	CTGCCAGCAATGTCGCTTCAAG
	Reverse Primer	TGGCTGCTCAACTGGTTGTTGG
PER1	Forward Primer	GCTTCAGAGATCTTGGCAGG
	Reverse Primer	GAGGGCACAGGTGAAGGATG
DBP	Forward Primer	TGACCCTCGGAGACACCGCT
	Reverse Primer	CACCACCTCCTGCCGCAACA
GAPDH	Forward Primer	AGGGCTGCTTTTAACTCTGGT
	Reverse Primer	CCCCACTTGATTTTGGAGGGA

### 2.10 Wheel running analysis of the rest/activity cycles

The mice were randomly divided into six groups (*n* = 9): ZT4 saline, ZT4 ciprofol, ZT4 propofol, ZT16 saline, ZT16 ciprofol and ZT16 propofol. They were acclimated to running wheels under LD (Light: Dark) conditions (8:00–20:00) 1 week before administration. On the 8^th^ day, single tail vein injections were administered at ZT4 and ZT16 (Saline: 2.75 mL/kg; Ciprofol: 5.5 mg/kg; Propofol: 11.5 mg/kg). And light condition was changed to constant darkness after administration in the second week with other environmental conditions (e.g., food and water availability, drug administration…) unchanged. Data were collected and analyzed using the ClockLab system after the continuous recording of running wheel activity for 2 weeks.

### 2.11 Statistical analysis

Data were processed and analyzed using Analyst Software 1.6.3 software, Excel, the JTK cycle package, the ClockLab system (version 6.1.15), and GraphPad Prism 9. Statistical analyses were conducted using unpaired *t*-tests, paired *t*-tests, one-way analysis of variance (ANOVA) (parametric test and Tukey’s multiple comparison test), one-way ANOVA with Dunn’s multiple comparison test, or the two-stage linear step-up procedure of the Benjamini, Krieger, and Yekutieli test. Unless specified otherwise, all data were presented as means ± SEM with the n value indicated for each dataset. Comparison of mean differences between two groups was conducted using independent-sample *t*-tests or one-way ANOVA with Dunn’s multiple comparison tests or with a two-stage linear step-up procedure of the Benjamini, Krieger, and Yekutieli test. The significance threshold was set at 0.05, two-tailed (not significant, ns; *p* > 0.05; *p < 0.05; **p < 0.01; ***p < 0.001; ****p < 0.0001). Mice from different litters were randomly assigned to different treatment groups, with no additional randomization used for the animal studies.

## 3 Results

### 3.1 Diurnal sedation effect of ciprofol and propofol

**Figure 1:**
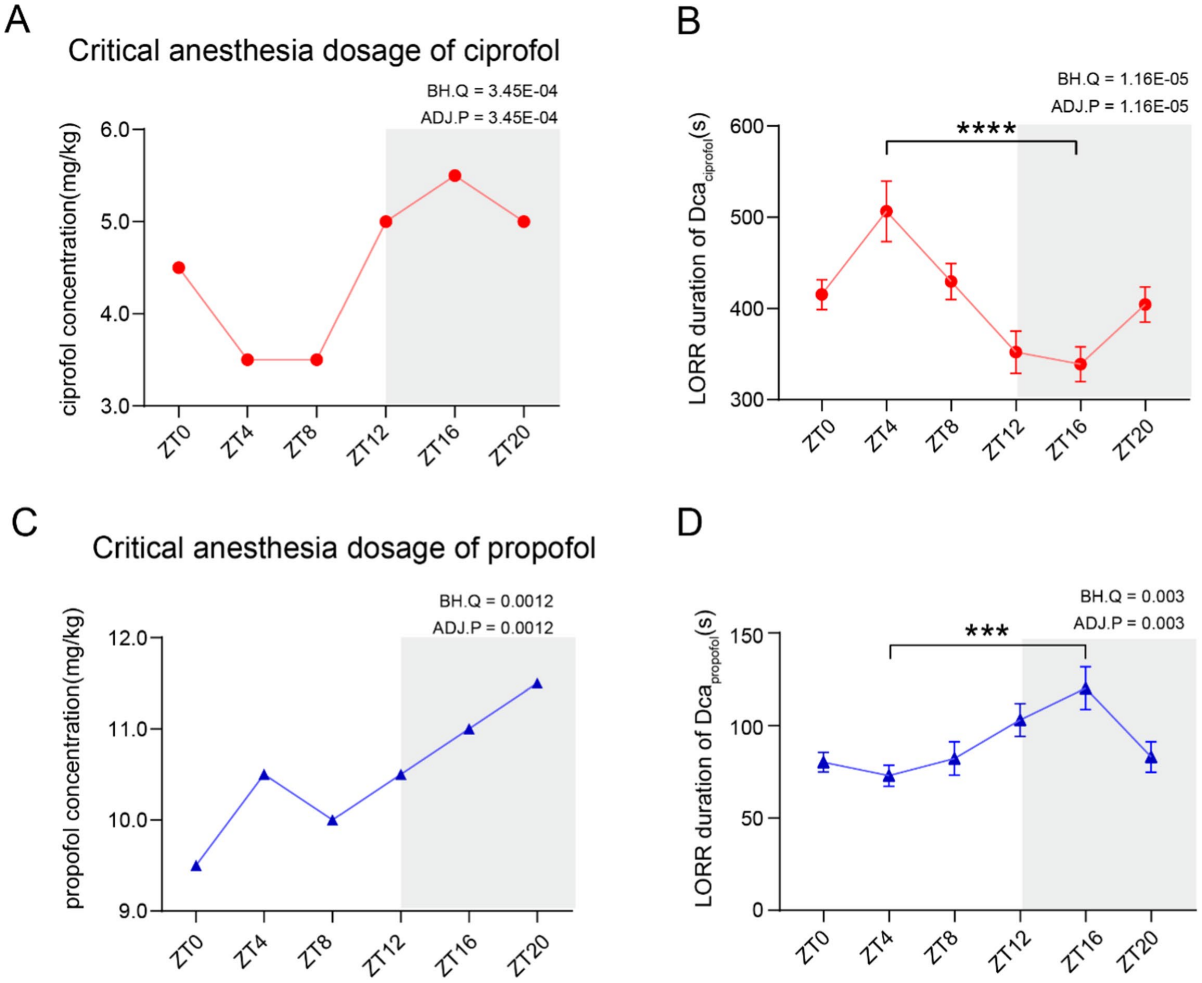
Critical anesthesia dosage of ciprofol and propofol follows a circadian variation. **(A)** Critical anesthetic dosage (D_ca_) of ciprofol follows a circadian rhythm (*n* = 7). **(B)** LORR duration of the highest D_ca_ requirement within a day. Ciprofol (5.5 mg/kg) showed a circadian difference (*n* = 10). **(C)** D_ca_ of propofol showed a circadian variation (*n* = 7). **(D)** LORR duration of the highest D_ca_ requirement within a day. Propofol (11.5 mg/kg) showed a circadian difference (*n* = 9). Circadian analyses were conducted using JTK_CYCLE (Hughes et al., 2010). Statistical analyses were conducted using unpaired unpaired *t*-tests. Data are presented as Means ± SEM. *p < 0.05, **p < 0.01, ***p < 0.001, ****p < 0.0001, ns *p* > 0.05.

To comprehensively investigate whether the anesthetic requirements follow a diurnal pattern, the D_ca_ of ciprofol and propofol was measured every 4 h throughout the day. The results demonstrated that the D_ca_ for both ciprofol and propofol exhibited a diurnal pattern (Figure 1A, ADJ.P_ciprofol_ = 3.45E-04, *n* = 7; Figure 1C, ADJ.P_propofol_ = 0.0012, *n* = 7). The highest D_ca_ for ciprofol was observed at ZT16 (5.5 mg/kg), while the lowest occurred at ZT4 and ZT8 (3.5 mg/kg). For propofol, the highest D_ca_ was at ZT20 (11.5 mg/kg) and the lowest at ZT0 (9.5 mg/kg). Overall, the anesthetic requirement was significantly higher during the dark phase than that during the light phase.

To assess the diurnal patterns in onset latency and loss of righting reflex (LORR) duration, the highest required D_ca_ (ciprofol: 5.5 mg/kg, propofol: 11.5 mg/kg) was administered to mice and the anesthetic effects were recorded. LORR typically begins immediately after the intravenous administration of ciprofol or propofol. If LORR does not occur, the mice will not return to this state in future tests (video in Table 3). Additionally, the LORR duration for both drugs showed significant diurnal variations (ADJ.P_ciprofol_ = 1.16E-05, *n* = 10, Figure 1B) (ADJ.P_propofol_ = 0.003, *n* = 9, Figure 1D). Further analysis revealed that the longest LORR duration of ciprofol (5.5 mg/kg) was recorded at ZT4, lasting 506.5 ± 33.11 s (Means ± SEM), while the shortest was at ZT16, lasting 338.8 ± 19.06 s (*p* < 0.0001). Conversely, propofol showed the shortest duration at ZT4, lasting 72.89 ± 5.64 s (Means ± SEM), and the longest at ZT16, lasting 120.20 ± 11.57 s (Means ± SEM, *p =* 0.0002) (Figure 1D). The LORR duration of ciprofol was significantly longer than that of propofol at both ZT4 and ZT16 (Supplementary Figure S1A).

**Table 3:** Raw data and video links.

Disposable data	
Excel	https://data.mendeley.com/preview/vyyhb2pkw9?a=66af9509-5554-4e66-9a5b-9079fcc005b8
Video	https://data.mendeley.com/preview/5txnzysh9s?a=a6ff6e32-e452-4a7d-9596-d44a65890d44
Western Blot file	https://data.mendeley.com/preview/t924myv8mh?a=76fd19e3-1c56-4af2-ad3c-6e642139322f

In summary, both the effective concentration and anesthesia effects of ciprofol and propofol followed a diurnal pattern, with higher requirements typically observed during the dark phase. Further investigations are needed to determine whether these rhythmic effects are attributable to metabolic processes or specific mechanisms of action at their target sites.

### 3.2 Distribution of ciprofol and propofol

To investigate whether the temporal pharmacological effects of ciprofol and propofol are caused by rhythmic changes in pharmacokinetics, drug distribution was analyzed based on the highest D_ca_ requirements. As the drugs were injected directly into the bloodstream, primarily exerting their anesthetic effects in the brain and being metabolized by the liver, drug concentrations were measured in these tissues during the LORR duration.

**Figure 2:**
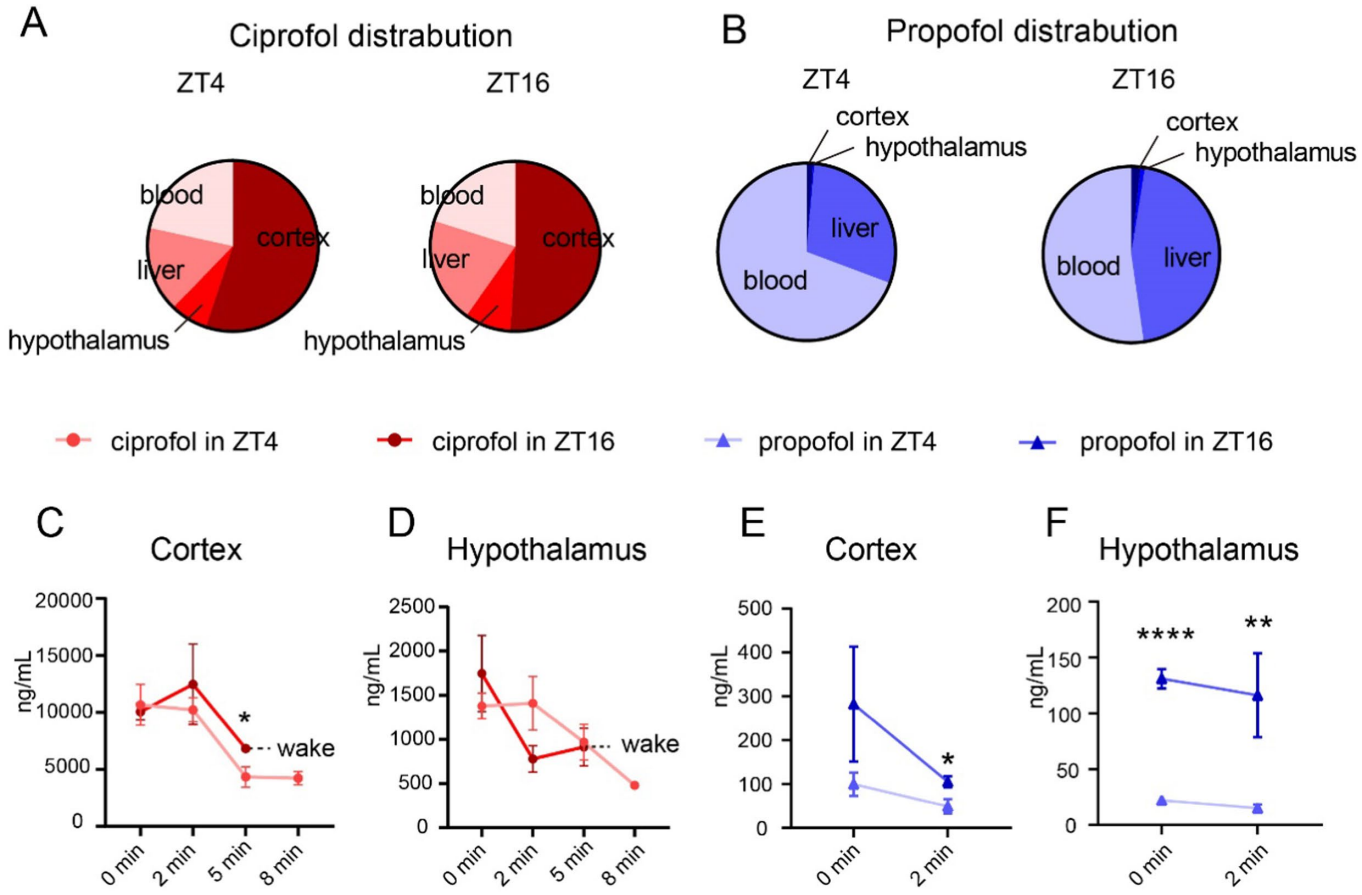
Distribution of ciprofol and propofol in cortex and hypothalamus. **(A)** Proportion of ciprofol distribution at ZT4 and ZT16 (ciprofol: 5.5 mg/kg). **(B)** Proportion of propofol distribution at ZT4 and ZT16 (propofol: 11.5 mg/kg). **(C,D)** Ciprofol concentration in cortex and hypothalamus 0, 2, 5, and 8 min after LORR at ZT4 and ZT16, respectively (*n* = 4–5). **(E,F)** Propofol concentration in cortex and hypothalamus 0 and 2 min of after LORR at ZT4 and ZT16, respectively (*n* = 4–5). Statistical analyses were conducted using unpaired unpaired *t*-tests. Data are presented as Means ± SEM. *p < 0.05, **p < 0.01, ***p < 0.001, ****p < 0.0001, ns *p* > 0.05.

Upon induction of LORR, ciprofol was primarily distributed in the cortex (55.09% at ZT4 and 50.93% at ZT16) (Figure 2A), whereas propofol was mainly distributed in the blood (69.30% at ZT4 [Figure 2B, left] and 52.26% at ZT16 [Figure 2B, right]).

Further analyses were conducted to understand diurnal differences in drug distribution. Generally, drug concentrations in each tissue gradually decreased over time until the mouse awoke. The mice in the ciprofol group typically regained consciousness within 8 min in ZT16, leading to a value of null at that time point (Figures 2C,D and Supplementary Figures S2A,B). The same situation applies to the values at 5 and 8 min in the propofol group were null for both ZT4 and ZT16 (Figures 2E,F) (Supplementary Figures S2C,D).

Notably, ciprofol concentration remained higher in the cortex 5 min after injection in ZT16 than in ZT4 (ZT4_5 min_ = 4335.00 ± 897.70 ng/mL [Means ± SEM], ZT16_5 min_ = 6818.00 ± 249.30 ng/mL [Means ± SEM], *p* = 0.0373), although initial concentrations were not significantly different (Figure 2C). The levels of ciprofol in the hypothalamus remained consistent throughout (Figure 2D). Additionally, ciprofol concentrations in the liver and blood were similar (Supplementary Figure S1).

For propofol, higher concentrations were consistently observed in the cortex and hypothalamus at ZT16 than at ZT4 (Figures 2E,F). Detailed analysis showed a difference in propofol levels in the cortex at 2 min (ZT4 = 49.13 ± 16.36 ng/mL [Means ± SEM], ZT16 = 105.40 ± 12.46 ng/mL [Means ± SEM], *p* = 0.034, Figure 2E). In the hypothalamus, propofol concentrations were significantly higher at ZT16 from the start than at ZT4 (ZT4_0 min_ = 22.23 ± 2.77 ng/mL [Means ± SEM], ZT16_0 min_ = 131.00 ± 8.54 ng/mL [Means ± SEM], *p* < 0.0001) and persisted for 2 min (Figure 2F).

Lipophilicity, an important physicochemical property of drugs, significantly influences the distribution and metabolism of various compounds. The lipid water partition coefficient (CLogP) for propofol was 3.929, while that for ciprofol was 4.373 (Sousa da Silva and Vranken, 2012). Consequently, ciprofol, which exhibits higher lipophilicity, more effectively crosses the blood–brain barrier (BBB) and is distributed extensively within the brain. Furthermore, ciprofol demonstrates stereoselectivity at the GABAA receptor owing to its R-type chiral molecular configuration, while the t-butylbicyclophosphorothionate (TBPS) inhibition index of propofol at GABA receptors is reportedly 10 (Zhou et al., 2024; Qin et al., 2017), indicating that propofol has little propensity to bind to these receptors. Therefore, propofol quickly binds to receptors in the brain and is metabolized via the bloodstream in the liver. Our findings align with previous research, showing that ciprofol mainly accumulates in the brain, whereas propofol is more abundant in the liver. These findings suggest that the circadian rhythm of ciprofol may be mainly influenced by brain activity, whereas that of propofol is more likely to be affected by liver metabolism. To investigate differences in the effects of these two drugs, we assessed the expression of brain receptors and metabolic enzymes in the peripheral tissues.

### 3.3 Metabolic enzyme levels in light and dark phases

To further confirm that the pharmacokinetics exhibit a diurnal rhythm, the expression of metabolic enzymes at ZT4 and ZT16 was compared.

**Figure 3:**
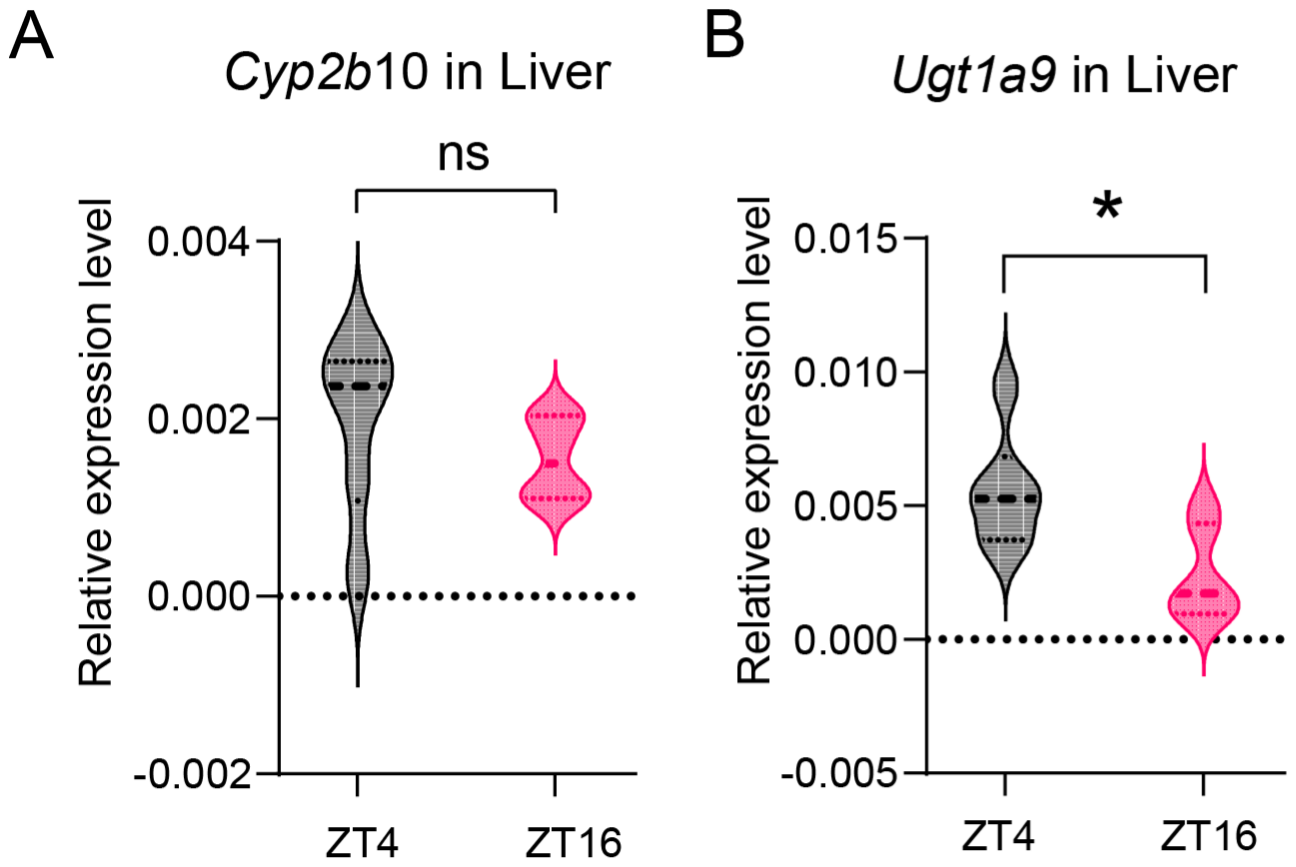
Diurnal variations in metabolic enzyme level in liver. **(A)** *Cyp2b6* mRNA expression level in the liver at ZT4 and ZT16 (*n* = 6). **(B)** *Ugt1a9* mRNA expression level in the liver at ZT4 and ZT16 (*n* = 6). Statistical analyses were conducted using unpaired *t*-tests. Data are presented as Means ± SEM. *p < 0.05, **p < 0.01, ***p < 0.001, ****p < 0.0001, ns *p* > 0.05.

Numerous studies have reported that CYP450 is predominantly involved in the oxidation of propofol by the liver (Oda et al., 2001), which is also a metabolic enzyme for ciprofol (Lu et al., 2024). Enzyme levels were measured in the livers of mice at ZT4 and ZT16. The mRNA level of *Cyp2b10* at ZT4 did not differ significantly from those at ZT16 (Figure 3A). These results suggest that diurnal variations in propofol efficacy may not be attributed to *Cyp2b10* expression.

Furthermore, propofol is metabolized by the UGT1A9 enzyme, which also contributes to the metabolism of ciprofol (Zhou et al., 2024; Xu et al., 2020). Our results indicate that *Ugt1a9* expression in the liver was higher at ZT4 than that at ZT16 (*p* = 0.019) (Figure 3B).

As ciprofol is predominantly distributed in the brain rather than the liver (Liao et al., 2022), brain receptors may have a more significant impact on the circadian rhythm than the UGT1A9 enzyme. We further investigated whether GABAAR levels also displayed diurnal features.

### 3.4 GABAAR subunit expression levels in light and dark phases

**Figure 4:**
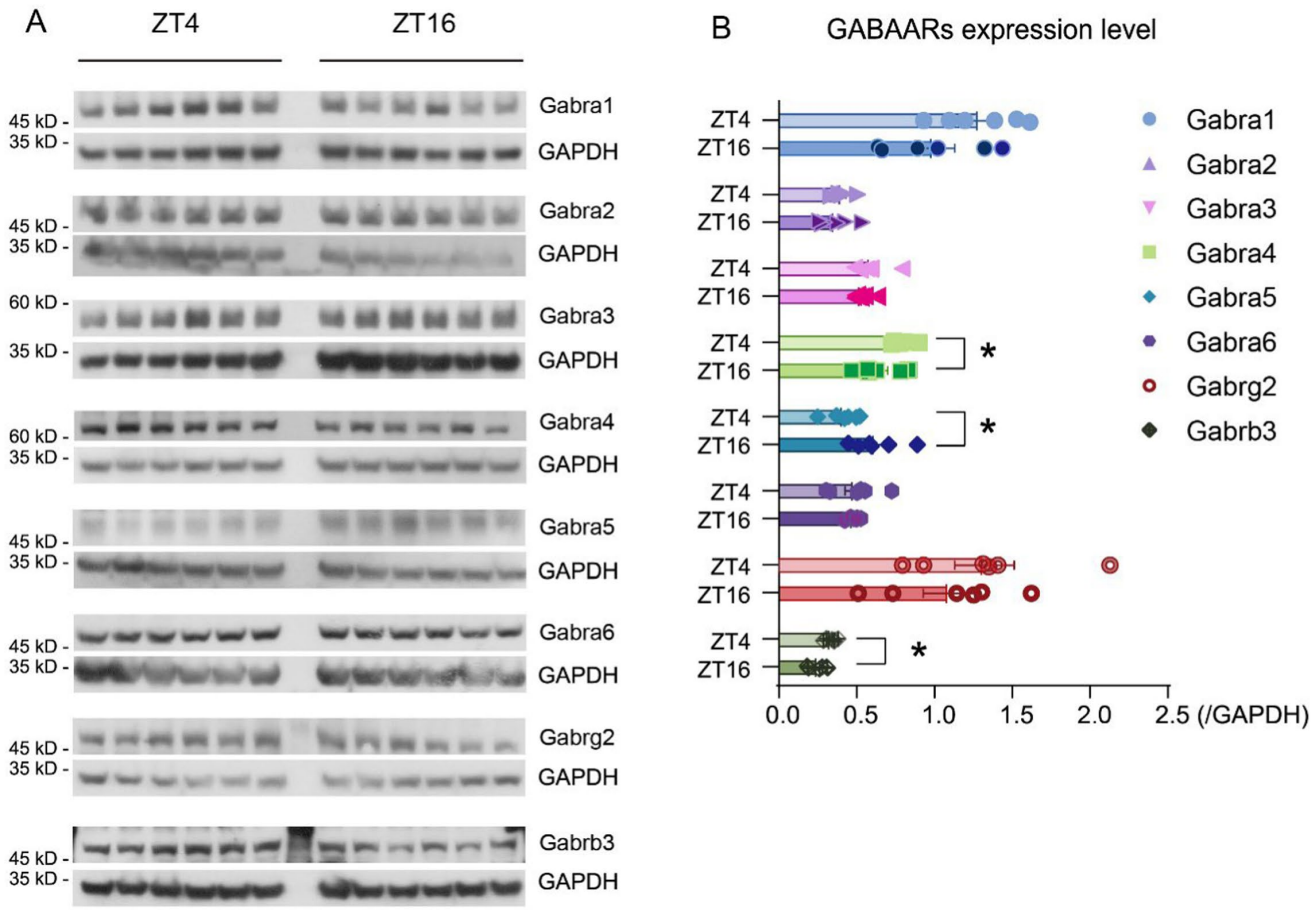
GABAAR subunit expression levels vary at ZT4 and ZT16. **(A)** Expression level of Gabra1-6, Gabrg2, Gabrb3, and Gapdh at ZT4 and ZT16 (*n* = 6). **(B)** Expression ratio of Gabra1-6, Gabrg2, Gabrb3, and Gapdh at ZT4 and ZT16 (expression ratio = expression level_GABAAR subunits_ / expression level_Gapdh_) (*n* = 6). Statistical analyses were conducted using unpaired unpaired *t*-tests. Data are presented as Means ± SEM. *p < 0.05, **p < 0.01, ***p < 0.001, ****p < 0.0001, ns *p* > 0.05.

It is widely acknowledged that both propofol and ciprofol exert anesthetic effects by acting on GABAARs in the brain (Qin et al., 2017; Jayakar et al., 2014). To explore if the expression of these receptors follows a diurnal pattern, the levels of GABAAR subunits were measured at ZT4 and ZT16 (Supplementary Figure S3). Our results showed that the α_4_ (Gabra4), α_5_ (Gabra5), and β_3_ (Gabrb3) subunits exhibited significant differences in expression at ZT4 and ZT16 (Figures 4A,B*, p* < 0.05). Specifically, α_4_ and β_3_ subunits were higher at ZT4 (Figure 4B), while α_5_ levels were lower at ZT4 (Figure 4B).

To verify whether the observed difference in receptor levels was due to transcriptional changes, real-time PCR was used to detect GABAAR subunit expression. Results indicate that *Gabra4* (translated into Gabra4) levels in the brain were higher at ZT4 than those at ZT16 (ZT4 = 1.96 ± 0.18, ZT16 = 1.58 ± 0.10, [Means ± SEM], *p* = 0.047) (Supplementary Figure S3D). Similarly, *Gabrb3* (translated into Gabrb3) levels were higher at ZT4 than those at ZT16 (ZT4 = 4.33 ± 0.16, ZT16 = 3.38 ± 0.20, [Means ± SEM], *p* = 0.004) (Supplementary Figure S3H). These expression differences are consistent with the protein levels observed. Although *Gabra5* (translated into Gabra5) did not show a statistically significant difference, there was a trend suggesting higher levels at ZT16 (*p* = 0.062), consistent with the difference in protein expression (Supplementary Figure S3E).

The β_3_ subunit is associated with sedation, hypnosis, and immobility (Weir et al., 2017), features of LORR induced by ciprofol and propofol. This implies that the higher β_3_ subunit levels at ZT4 may contribute to a longer duration of ciprofol effects during this time, as previously suggested. Given the circadian differences observed in certain side-effect-related subunits, such as α_5_ (Antkowiak and Rudolph, 2016), we further investigated whether the side effects of propofol exhibited diurnal features and whether ciprofol was similarly affected.

### 3.5 Molecular modeling of the binding of ciprofol/propofol to GABRB3

To demonstrate the differences in circadian pattern of anesthetic effects between ciprofol and propofol, we hypothesized that the rhythmic anesthetic effect of ciprofol depends more on the diurnal expression of the target protein GABRB3 due to its wide distribution in the brain and overall higher affinity to GABA receptors according to radioligand binding assays (Qin et al., 2017). However, there are few structural investigations on the binding pose of ciprofol to a specific GABAA receptor, nor are there binding affinity comparisons between ciprofol and propofol to specific receptors.

**Figure 5:**
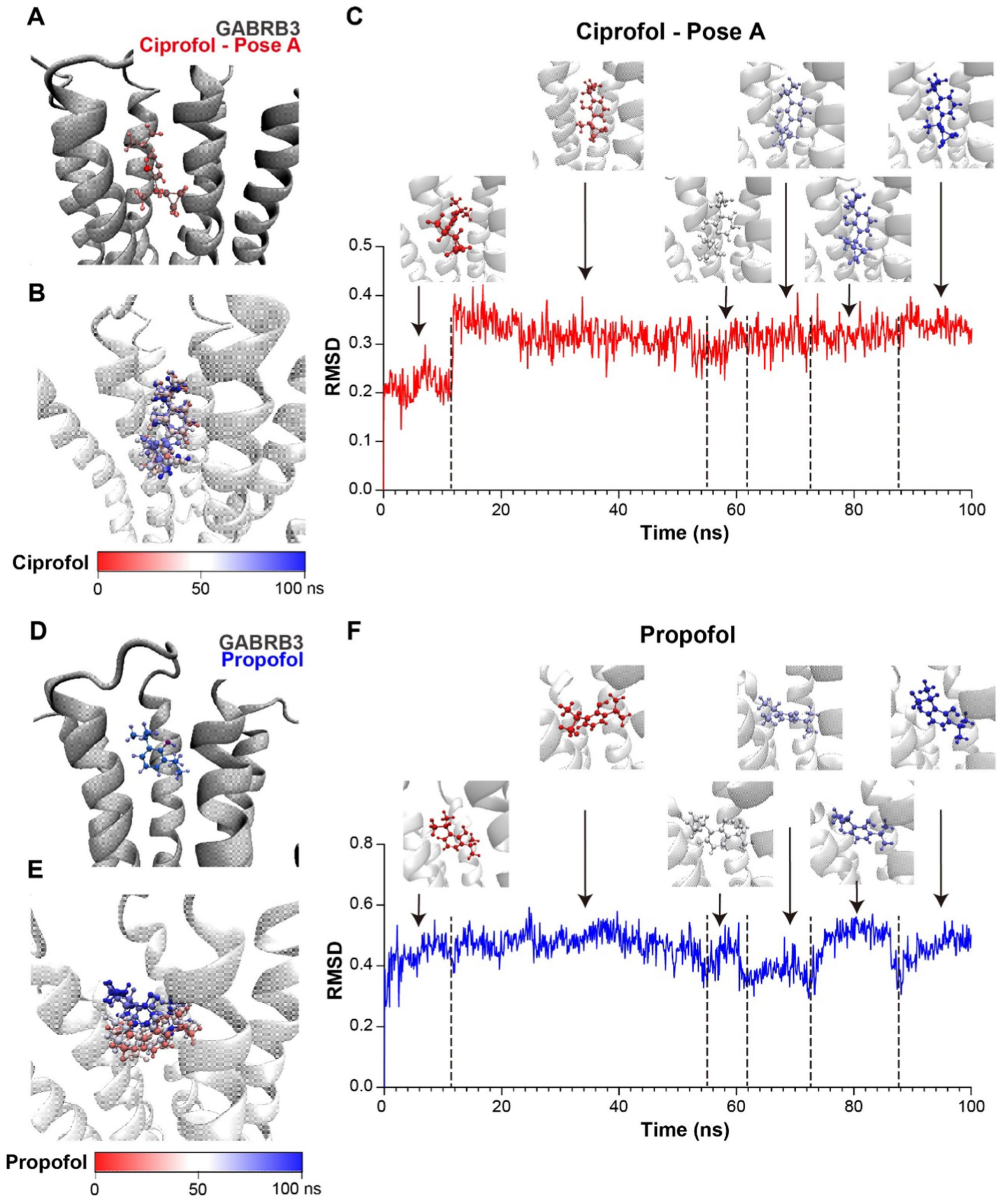
Ciprofol exhibits a stronger binding affinity to the GABRB3. **(A)** Structure model of ciprofol (pose A, red) and GABRB3 dimer (gray). **(B)** Conformations of ciprofol (pose A) during molecular dynamics simulation, presented by timestep. (Red to blue, 0–100 ns.) **(C)** RMSD of ciprofol during simulation and ciprofol conformations at respective stage. (Red to blue, 0–100 ns.) **(D)** Structure model of propofol (blue) and GABRB3 dimer (gray). The orange circles indicated the position of cyclopropyl. **(E)** Conformations of propofol during molecular dynamics simulation, presented by timestep. (Red to blue, 0 ns to 100 ns.) **(F)** RMSD of propofol during simulation and propofol conformations at respective stage. (Red to blue, 0 ns to 100 ns).

Consequently, to confirm our hypothesis, we carried out molecular modeling according to the existed CryoEM structures of GABRB3 (PDB ID: 7A5V) and propofol with GABA receptor α_1_β_2_γ_2_ subtype (PDB ID: 6X3T). Since GABA receptors have stereoselectivity to ciprofol, and R-ciprofol was reported to have a better effect, R-ciprofol was used for molecular dynamics analysis. To alter propofol to R-ciprofol in PDB 6X3T, there are two possible pose-downward cyclopropyl (Figure 5A, Pose A) and upward cyclopropyl (Supplementary Figure S4A, Pose B). For GABA receptors are highly conserved at the helical bundle (Supplementary Figure S4A) and α_1_, β_2/3_, and γ_2_ subunits are the most common subunits found in different brain regions (Waldvogel, 2021), we aligned the two reference structures and moved the small molecules (propofol, ciprofol pose A and ciprofol pose B) to GABRB3 dimer, respectively, (Figures 5A,D and Supplementary Figure S4B). After energy minimization, a 100-ns molecular dynamics simulation was carried out to investigate the binding pose of propofol/ciprofol to GABRB3 and for further binding energy calculation.

According to the trajectory clustering and different stages of root mean square deviation (RMSD), ciprofol pose B could even rotate to pose A during the simulation (Supplementary Figures S4C,D), indicating the unstable binding of this pose. On the contrary, pose A exhibited a stable binding pattern, so we chose it for binding affinity calculation via gmx_MMPBSA (Miller et al., 2012; Valdés-Tresanco et al., 2021). The RMSD of the small molecules could reflect the binding mode at GABRB3 pocket. Cipofol pose A is the most stable, the conformation was convergent in less than 20 ns (Figures 5B,C). While propofol, also adopted different binding modes (Figures 5E,F) during the simulation. The last 20 ns of the simulation were separated to 200 frames for binding energy calculation, and the binding energy of ciprofol is −10.4 ± 2.48 kcal/mol, while propofol is −5.89 ± 3.73 kcal/mol (Supplementary Table S1), indicating a higher binding affinity of R-ciprofol to GABRB3 compared with propofol, further confirming our hypothesis.

The computational results provided possible binding conformation of ciprofol to GABRB3, and demonstrated a more stable interaction between ciprofol and GABRB3 compared with propofol, supporting our experimental data.

### 3.6 Locomotor impairment and depression induced by propofol in the light phase

**Figure 6:**
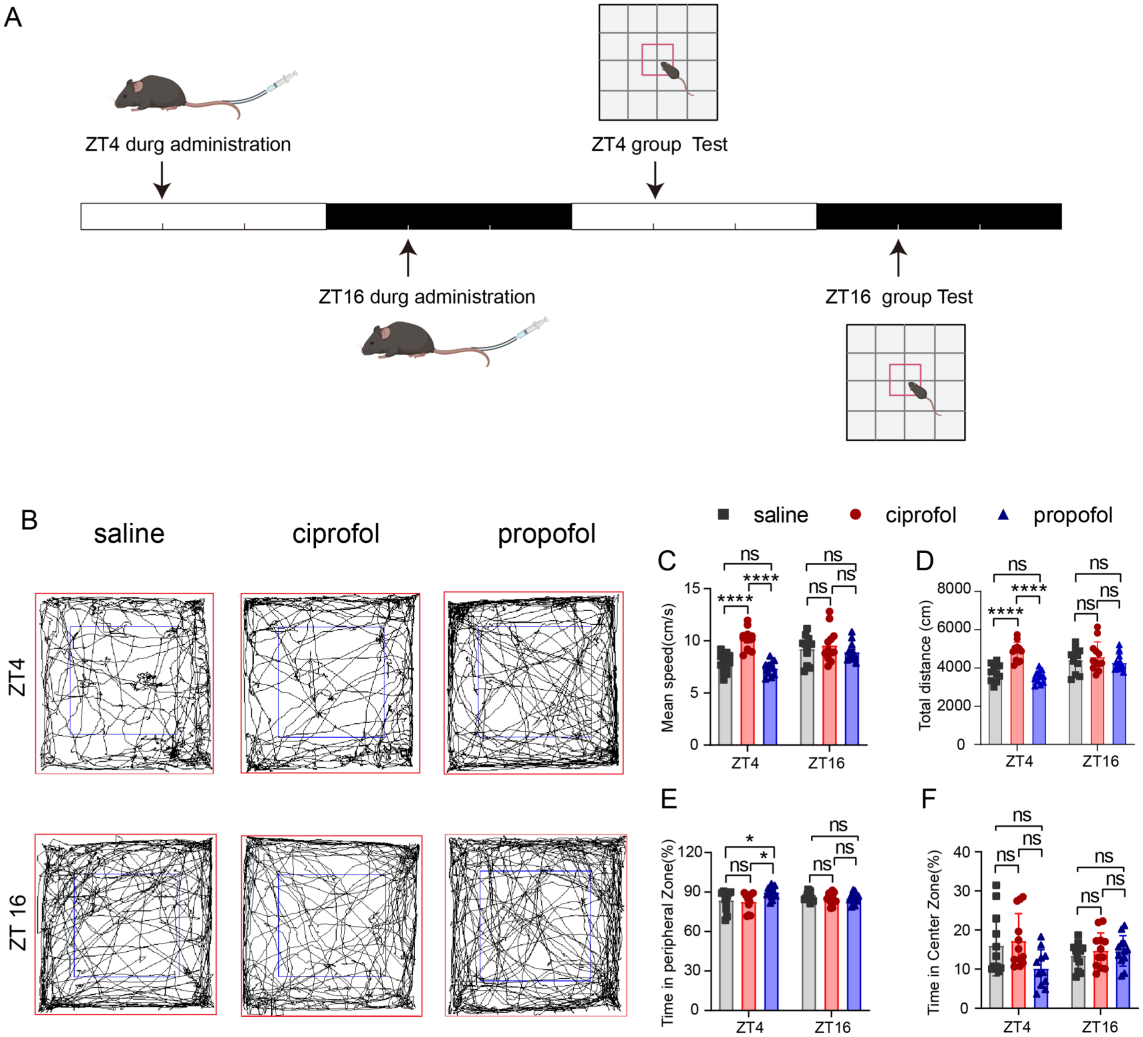
Effects of ciprofol and propofol on locomotion and mood in 24 h of ZT4 and ZT16 treatments. **(A)** Experimental scheme diagram. **(B)** Movement and activity trajectories of mice at 24 h after injection of saline (2.75 mL/kg, equal volume of ciprofol), ciprofol (5.5 mg/kg) and propofol (11.5 mg/kg) in ZT4 and ZT16 (*n* = 12). **(C–F)** Mean speed, total travel distance, time in the peripheral and central zones after saline administration (2.75 mL/kg, equal volume of ciprofol), ciprofol (5.5 mg/kg) and propofol (11.5 mg/kg) in ZT4 and ZT16, respectively (*n* = 12). Statistical analyses were conducted using one-way ANOVA with two-stage linear step-up procedure of Benjamini, Krieger and Yekutieli test. Data are presented as Means ± SEM. *p < 0.05, **p < 0.01, ***p < 0.001, ****p < 0.0001, ns *p* > 0.05.

To elucidate the circadian differences in the side effects on locomotor activity and depression between propofol and ciprofol, mice were injected at ZT4 and ZT16, followed by an open-field test (OPT) (Figure 6A).

Results suggest that ciprofol administration at ZT4 significantly increased locomotor speed compared to both saline group and propofol group (Speed_saline_ = 7.89 ± 0.26 cm/s, Speed_ciprofol_ = 10.12 ± 0.29 cm/s, Speed_propofol_ = 7.37 ± 0.19 cm/s [Means ± SEM], *p* < 0.0001, Figures 6B,C). In contrast, at ZT16, neither propofol nor ciprofol impaired locomotion (*p* > 0.05). Additionally, the total distance also increased in the ciprofol group at ZT4, while propofol did not (Figure 6D). And at ZT16, both propofol and ciprofol did not affect the total distance (Figure 6D), indicating that compared with proporol, ciprofol has an excitatory effect on locomotion during the resting phase.

Moreover, in the OPT assay, propofol caused mice to stay more in the peripheral zone at ZT4 (Time ratio_saline_ = 83.96 ± 2.24%, Time ratio_ciprofol_ = 82.74 ± 2.01%, Time ratio_propofol_ = 90 ± 1.32%, [Means ± SEM], *p_saline vs ciprofol_* = 0.6518, *p*_ciprofol vs propofol_ = 0.0108, F (DFn, DFd) = 0.3863) (Figures 6E,F), indicating a tendency for propofol to induce depressive effects during the resting phase. These results indicate that ciprofol does not induce depressive effects when administered at any time, making it superior to propofol in this regard.

We also conducted the OPT for a duration of 3 h at ZT4 and ZT16 (Supplementary Figure S5A) and found that the propofol group exhibited decreased mean speed and total distance at ZT4 compared to the ciprofol group (Speed_ciprofol_ = 8.88 ± 0.49 cm/s, Speed_propofol_ = 6.93 ± 0.48 cm/s [Means ± SEM], *p* = 0.0042) (Total distance_ciprofol_ = 4,262 ± 234.7 cm, Total distance_propofol_ = 3,330 ± 231.3 cm) (Supplementary Figures S5C,D). Time in the peripheral and central zones did not show significant differences during this relatively short period (Supplementary Figures S5E,F).

These results suggest that propofol is more likely to impair locomotion and induce depressive symptoms during the light phase than dark phase, while ciprofol slightly increases locomotor activity and reduces depression in the same term.

### 3.7 Cognitive and memory impairment induced by propofol in the dark phase

**Figure 7:**
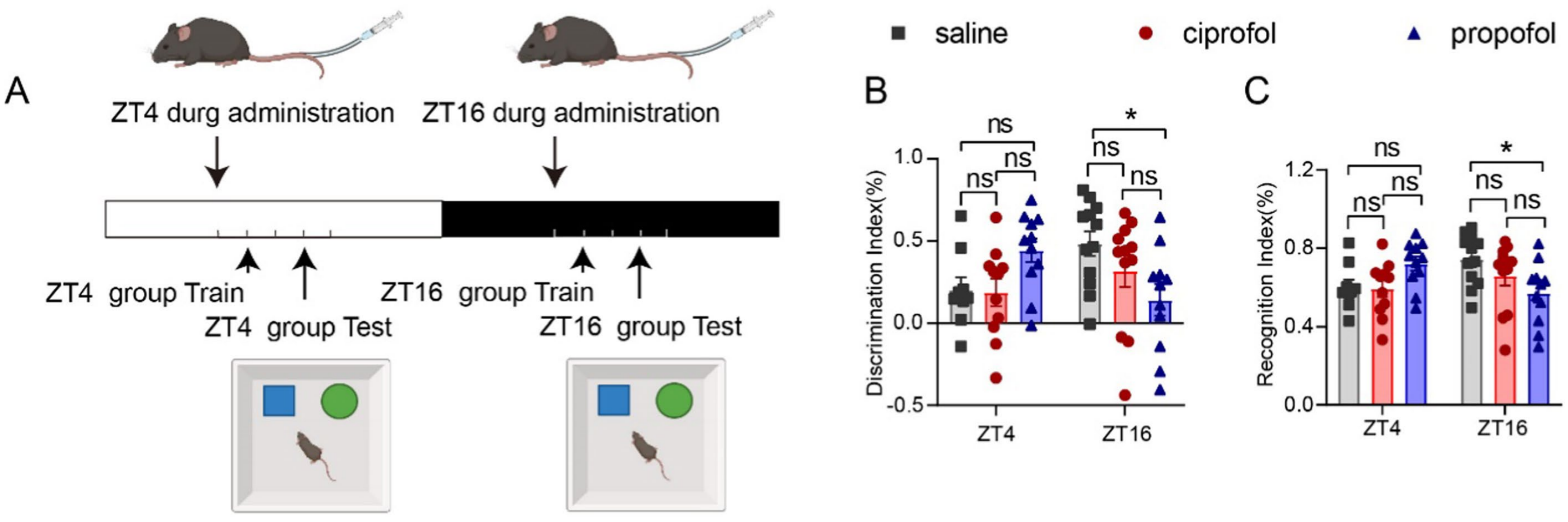
Effects of ciprofol and propofol on cognitive and memory ability in 3 h of ZT4 and ZT16 treatments. **(A)** Experimental scheme diagram. The familiar period in 1 h, followed by the test period of the new and old object recognition experiment in 3 h (saline [2.75 mL/kg, equal volume of ciprofol], ciprofol [5.5 mg/kg] and propofol [11.5 mg/kg]). **(B)** Discrimination index (DI) and Recognition index (RI) measured 3 h after the drug administration at ZT4 and ZT16, respectively (*n* = 11–12). DI = (new - old)/(new + old). RI = new / (new + old). Statistical analyses were conducted using one-way ANOVA with two-stage linear step-up procedure of Benjamini, Krieger and Yekutieli test. Data are presented as Means ± SEM. *p < 0.05, **p < 0.01, ***p < 0.001, ****p < 0.0001, ns *p* > 0.05.

Propofol has been reported to induce cognitive dysfunction, particularly during surgical procedures (Liu et al., 2022) or at relatively higher anesthetic dosages (Sato et al., 2005). We primarily studied whether propofol and ciprofol would affect short-term cognitive function in mice using a novel object recognition test under a critical anesthetic dose. Familiarity periods were conducted 1 h after administration, and testing periods were conducted for 3 h (Figure 7A). Compared to the control group, the discrimination index (DI) in the ZT16 group significantly decreased following propofol injection in 3 h (DI_saline_ = 0.48 ± 0.07%, DI_propofol_ = 0.14 ± 0.09%, [Means ± SEM], *p* = 0.0254) (Figure 7B). In contrast, no significant difference was observed at ZT4, indicating that propofol can impair cognitive and memory abilities when administered during the dark phase.

The recognition index (RI) exhibited a similar diurnal pattern to the DI at ZT16 (RI_saline_ = 0.74 ± 0.03%, RI_propofol_ = 0.57 ± 0.04%, [Means ± SEM], *p* = 0.0254) (Figure 7C). There were no statistically significant differences in the DI or RI for the ciprofol group compared to the saline group (Figures 7B,C, *p* > 0.05). Thus, ciprofol is superior to propofol regarding its impact on cognitive function.

To assess the duration of cognitive and memory impairment induced by propofol, the measurements were repeated at the 24th hour (Supplementary Figure S6A). Fortunately, the cognitive impairment caused by propofol at D_ca_ doses expired at this point (Supplementary Figures S6B,C, *p* > 0.05).

### 3.8 Disturbance of ciprofol and propofol on circadian rhythm

**Figure 8:**
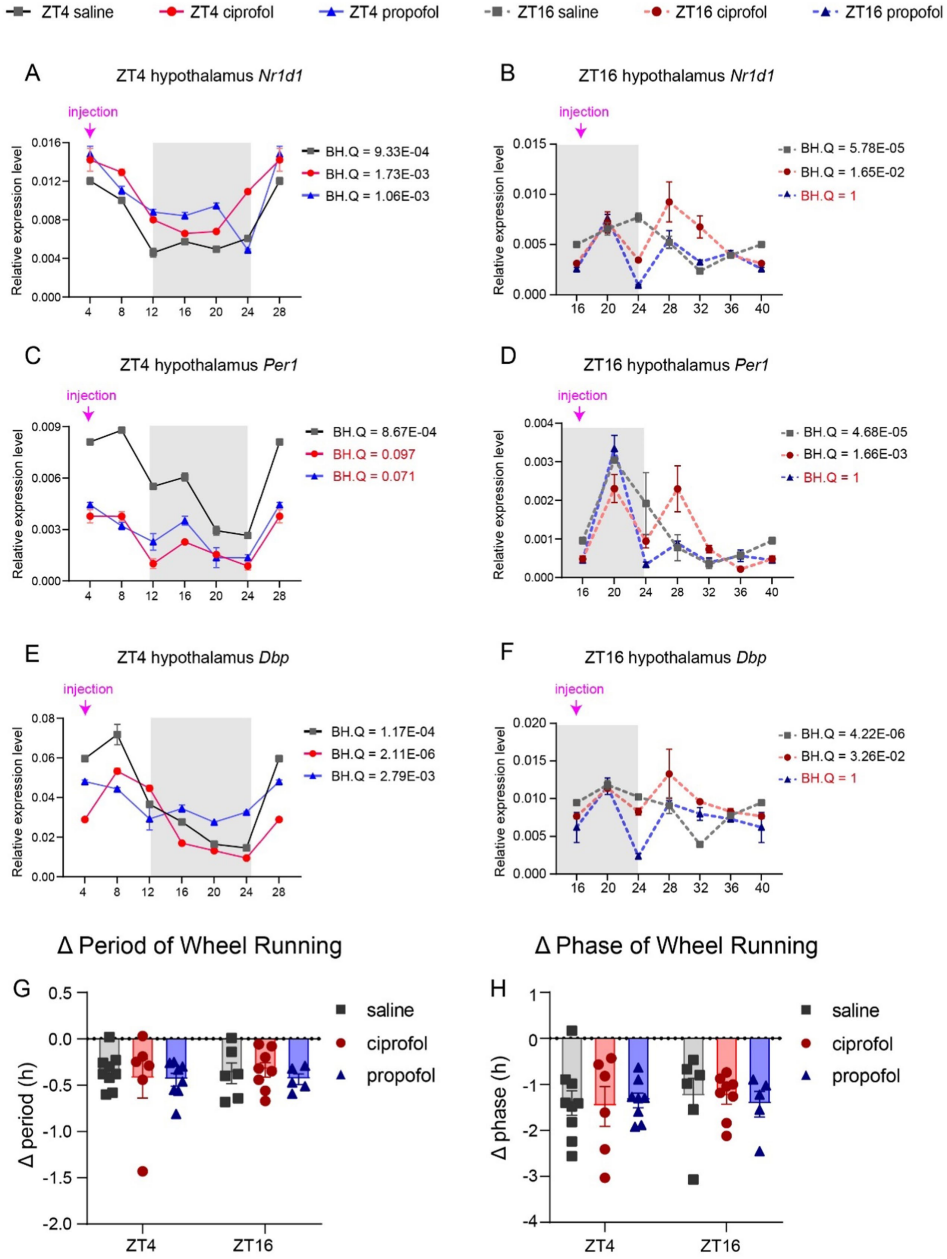
Disturbance of ciprofol and propofol on circadian genes and rest/activity rhythm. **(A)** *Nr1d1* gene expression levels 24 h after saline (2.75 mL/kg), ciprofol (5.5 mg/kg), and propofol (11.5 mg/kg) injected at ZT4 in the hypothalamus (*n* = 3). **(B)** *Nr1d1* gene expression levels 24 h after saline (2.75 mL/kg), ciprofol (5.5 mg/kg), and propofol (11.5 mg/kg) injected at ZT16 in the hypothalamus (*n* = 3). **(C)** *Per1* gene expression levels 24 h after saline (2.75 mL/kg), ciprofol (5.5 mg/kg), and propofol (11.5 mg/kg) injected at ZT4 in the hypothalamus (*n* = 3). **(D)** *Per1* gene expression levels 24 h after saline (2.75 mL/kg), ciprofol (5.5 mg/kg), and propofol (11.5 mg/kg) injected at ZT16 in the hypothalamus (*n* = 3). **(E)** *Dbp* gene expression levels 24 h after saline (2.75 mL/kg), ciprofol (5.5 mg/kg), and propofol (11.5 mg/kg) injected at ZT4 in the hypothalamus (*n* = 3). **(F)** *Dbp* gene expression levels 24 h after saline (2.75 mL/kg), ciprofol (5.5 mg/kg), and propofol (11.5 mg/kg) injected at ZT16 in the hypothalamus (*n* = 3). **(G)** Period changes induced by saline (2.75 mL/kg), propofol (11.5 mg/kg), and ciprofol (5.5 mg/kg) at ZT4 and ZT16 (*n* = 4–9). (*Δ* period = period_LD_ – period_DD_). **(H)** Phase changes induced by saline (2.75 mL/kg), propofol (11.5 mg/kg), and ciprofol (5.5 mg/kg) at ZT4 and ZT16 (*n* = 4–9). (Δ phase = phase_LD_ – phase_DD_). Circadian analyses were conducted using JTK_CYCLE (Hughes et al., 2010). Statistical analyses were conducted using one-way ANOVA with two-stage linear step-up procedure of Benjamini, Krieger and Yekutieli test. Data are presented as Means ± SEM. *p < 0.05, **p < 0.01, ***p < 0.001, ****p < 0.0001, ns *p* > 0.05.

The anesthetic effects were clearly influenced by circadian rhythms (Antkowiak and Rudolph, 2016); however, it remains uncertain whether D_ca_ of anesthesia could also disrupt these rhythms. To investigate this, the expression levels of three representative genes were assessed in the hypothalamus, along with rest/activity patterns following D_ca_ drug injection at ZT4 and ZT16. The results indicated that, regardless of the timing of the injection, the saline group exhibited circadian rhythms in the expression of *Nr1d1*, *Per1*, and *Dbp* (Figure 8, BH.Q < 0.05). However, at ZT16, propofol significantly disrupted the circadian rhythms of *Nr1d1*, *Per1*, and *Dbp* (BH.Q = 1, Figures 8B,D,F). Additionally, the circadian feature of *Per1* at ZT4 was notably disturbed by propofol (BH.Q = 0.071) and ciprofol (BH.Q = 0.097) (Figure 8C). Propofol administered during the dark phase was more likely to influence the circadian rhythms of *Nr1d1* and *Dbp* in the hypothalamus. In contrast, ciprofol had a lesser impact on circadian disorders than propofol, suggesting that it is more supportive of circadian rhythm at the molecular level. Notably, the injection procedure was performed under dim light conditions, which may have introduced transient fluctuations in gene expression (Challet et al., 2003). This could partly explain the observed up-regulation of NR1d1, Per1, and Dbp at ZT20 in the ZT16 series. Nonetheless, when compared to the control group at the corresponding time points, the observed effects still provide meaningful insights into the drug’s impact on circadian rhythms.

These molecular effects may extend to physiological levels, prompting us to evaluate rest/activity rhythms after saline, propofol, and ciprofol injections at ZT4 and ZT16 through wheel running. The results showed that none of the three groups significantly affected activity/rest rhythms (Figures 8G,H, *p* > 0.05) (Supplementary Figure S7). These findings suggest that disruptions in circadian gene expression in the hypothalamus induced by anesthetics do not necessarily lead to dose-dependent physiological changes.

## 4 Discussion

This study primarily examined the anesthetic and side effects of ciprofol compared to propofol from a chronopharmacological perspective. Ciprofol demonstrates several clinical advantages over propofol, in terms of higher anesthesia success rate (Li et al., 2022), reduced injection pain and better cardiovascular stability (Yu et al., 2024). These properties make ciprofol a more favorable option for clinical use, especially in high-risk patient populations such as the elderly (Hudaib et al., 2024). However, comparative data on the longer-term and uncommon clinical side effects of these two drugs, such as anxiety and memory impairment, are still pending. Despite the scarcity of clinical reports addressing differences in anesthesia demand between day and night (Shen et al., 2021), such variations indeed exist—and this may similarly apply to the occurrence of side effects. Medication should adhere to the principles of chronopharmacology, avoiding the use of high-risk drugs in vulnerable populations and minimizing drug dosages at susceptible time points. Our findings demonstrated day-night differences in the anesthetic effects of ciprofol and propofol. Additionally, it further examined the day-night differences in their side effects, as well as their impacts on the expression of circadian genes and associated activities. This study contribute to the clinical optimization of anesthesia timing and broaden the options available for anesthetic agents in clinical practice. In addition, this research provides potential mechanisms and valuable insights that may inform future strategies for addressing diurnal variations in drug efficacy.

Previous studies have often focused on the depth and duration of anesthesia of intravenous anesthetic agents (Shen et al., 2021; Challet et al., 2007; Sato et al., 2005; Wang et al., 2020). In this study, a venous visualization injector was employed to facilitate tail vein injections, and statistical analysis was limited to mice that completed the injection in a single attempt. This approach yielded significant diurnal differences in other aspect of anesthesia effect ─ anesthesia demand, allowing for adjustments to dosage regimens based on diurnal patterns for more precise administration of anesthesia.

While propofol increased in lipophilicity and formed ciprofol, ciprofol was found to cross the blood brain barrier more effectively and accumulate to a greater extent than propofol (Qin et al., 2017; Liao et al., 2022). Furthermore in our study, the R-configured form of ciprofol exhibited significantly higher binding potency at the GABRB3 than propofol. Consequently, the diurnal rhythm of propofol was predominantly influenced by metabolic factors, while that of ciprofol was significantly affected by receptor expression. Additionally, the GABAAR β_3_ subunits are key targets for the anesthetic effects of both ciprofol and propofol (Kreuzer et al., 2020). The increased expression of these receptors corresponded with a longer duration of ciprofol during the light phase. Although the *Cyp2b10* level showed no diurnal rhythm in our study as reported in other studies (Viitala et al., 2001). UGT1A9, another propofol-related metabolic enzyme (Xu et al., 2020), showed a concordant diurnal pattern with LORR duration of propofol in our study.

The depression induced by propofol (Song et al., 2019; Chang et al., 2020), in our study was more likely to occur during the light phase than in dark phase, with the depressive effects becoming more pronounced at 24 h post-injection than at 3 h post-injection. We also observed rhythmic expression of the antidepressant-related GABAAR α_5_ subunit (Fee et al., 2021; Luscher et al., 2023), which decreased during the light phase. This diurnal variation in α_5_ subunit expression may contribute to the rhythmic nature of propofol-induced depression.

In this study, mice exhibited cognitive impairments 3 h after propofol injection, which aligns with the inherent characteristics of propofol associated with a_5_ subunit (Antkowiak and Rudolph, 2016; Zurek et al., 2014). These functional impairments were more likely to occur during the dark phase, coinciding with peak expression levels of α_5_ subunits.

The timing of anesthesia administration affects circadian rhythm (Mihara et al., 2012). In this study, propofol influenced the rhythm of the *Per1* gene at ZT4, similar to ciprofol, and also at ZT16, disrupting the rhythms of *Per1, Nr1d1*, and *Dbp*. Literature indicates that anesthetizing mice with propofol for 4 h does not alter their rest/activity rhythm (Mizuno et al., 2022), yet it can still alter rhythm genes. This suggests that the behavioral effects of propofol may be evident in other areas, such as sleep rhythms (Yin et al., 2022). Considering these alterations in circadian rhythm-related genes, propofol may be unsuitable for use during active phases.

## Data Availability

The datasets presented in this study can be found in online repositories. The names of the repository/repositories and accession number(s) can be found in the article/Supplementary material.

## References

[ref1] Antkowiak B. Rudolph U. (2016). New insights in the systemic and molecular underpinnings of general anesthetic actions mediated by gamma-aminobutyric acid a receptors. Curr. Opin. Anaesthesiol. 29, 447–453. doi: 10.1097/ACO.0000000000000358, PMID: 27168087 PMC4957807

[ref2] Berendsen H. J. C. van der Spoel D. van Drunen R. (1995). GROMACS: a message-passing parallel molecular dynamics implementation. Comput. Phys. Commun. 91, 43–56. doi: 10.1016/0010-4655(95)00042-E

[ref3] Challet E. Gourmelen S. Pevet P. Oberling P. Pain L. (2007). Reciprocal relationships between general (Propofol) anesthesia and circadian time in rats. Neuropsychopharmacology 32, 728–735. doi: 10.1038/sj.npp.1301081 16641940

[ref4] Challet E. Poirel V. J. Malan A. Pévet P. (2003). Light exposure during daytime modulates expression of Per1 and Per2 clock genes in the suprachiasmatic nuclei of mice. J. Neurosci. Res. 72, 629–637. doi: 10.1002/jnr.10616, PMID: 12749028

[ref5] Chang H. Li S. Li Y. Hu H. Cheng B. Miao J.. (2020). Effect of sedation with dexmedetomidine or propofol on gastrointestinal motility in lipopolysaccharide-induced endotoxemic mice. BMC Anesthesiol. 20:227. doi: 10.1186/s12871-020-01146-z, PMID: 32894042 PMC7487735

[ref6] Chowdhury D. Wang C. Lu A. P. Zhu H. L. (2019). Understanding quantitative circadian regulations are crucial towards advancing chronotherapy. Cells 8, 883–905. doi: 10.3390/cells8080883, PMID: 31412622 PMC6721722

[ref7] Fee C. Prevot T. D. Misquitta K. Knutson D. E. Li G. Mondal P.. (2021). Behavioral deficits induced by somatostatin-positive GABA neuron silencing are rescued by alpha 5 GABA-A receptor potentiation. Int. J. Neuropsychopharmacol. 24, 505–518. doi: 10.1093/ijnp/pyab002, PMID: 33438026 PMC8278801

[ref8] Fujimura A. Ushijima K. (2023). Understanding the role of chronopharmacology for drug optimization: what do we know? Expert. Rev. Clin. Pharmacol. 16, 655–668. doi: 10.1080/17512433.2023.2233438, PMID: 37403790

[ref9] Gaussian 16 Rev (2016). C.01 [program]. Wallingford, CT.

[ref10] Hudaib M. Malik H. Zakir S. J. Rabbani S. Gnanendran D. Syed A. R. S.. (2024). Efficacy and safety of ciprofol versus propofol for induction and maintenance of general anesthesia: a systematic review and meta-analysis. J. Anesth. Analg. Crit. Care 4, 26–36. doi: 10.1186/s44158-024-00160-8, PMID: 38605424 PMC11008023

[ref11] Hughes M. E. Hogenesch J. B. Kornacker K. (2010). JTK_CYCLE: an efficient nonparametric algorithm for detecting rhythmic components in genome-scale data sets. J. Biol. Rhythm. 25, 372–380. doi: 10.1177/0748730410379711, PMID: 20876817 PMC3119870

[ref12] Jayakar S. S. Zhou X. Chiara D. C. Dostalova Z. Savechenkov P. Y. Bruzik K. S.. (2014). Multiple propofol-binding sites in a γ-aminobutyric acid type a receptor (GABAAR) identified using a photoreactive propofol analog. J. Biol. Chem. 289, 27456–27468. doi: 10.1074/jbc.M114.581728 25086038 PMC4183786

[ref13] Kilicarslan G. Alkan M. Kurtipek O. Unal Y. Sivgin V. Dikmen K. (2021). The effect of circadian Rhytm in patients undergoing spinal anesthesia. Agri 33, 168–175. doi: 10.14744/agri.2021.65807, PMID: 34318918

[ref14] Kreuzer M. Butovas S. Garcia P. S. Schneider G. Schwarz C. Rudolph U.. (2020). Propofol affects Cortico-hippocampal interactions via beta3 subunit-containing GABA(a) receptors. Int. J. Mol. Sci. 21, 5844–5859. doi: 10.3390/ijms21165844, PMID: 32823959 PMC7461501

[ref15] Li J. Wang X. Liu J. Wang X. Li X. Wang Y.. (2022). Comparison of ciprofol (HSK3486) versus propofol for the induction of deep sedation during gastroscopy and colonoscopy procedures: a multi-Centre, non-inferiority, randomized, controlled phase 3 clinical trial. Basic Clin. Pharmacol. Toxicol. 131, 138–148. doi: 10.1111/bcpt.13761, PMID: 35653554 PMC9543620

[ref16] Li X. Yan L. Wang L. Chen H. Yang B. (2024). Study on the preventive effect of dexmedetomidine on anesthetic associated sleep disturbance in young to middle-aged female patients undergoing hysteroscopy: a study protocol for a crossover randomized controlled trial. Trials 25:480. doi: 10.1186/s13063-024-08311-6, PMID: 39010171 PMC11251345

[ref17] Liao J. Li M. Huang C. Yu Y. Chen Y. Gan J.. (2022). Pharmacodynamics and pharmacokinetics of HSK3486, a novel 2,6-Disubstituted phenol derivative as a general anesthetic. Front. Pharmacol. 13:830791. doi: 10.3389/fphar.2022.830791 35185584 PMC8851058

[ref18] Liu P. Zhao S. Qiao H. Li T. Mi W. Xu Z.. (2022). Does propofol definitely improve postoperative cognitive dysfunction?-a review of propofol-related cognitive impairment. Acta Biochim. Biophys. Sin. Shanghai 54, 875–881. doi: 10.3724/abbs.2022067, PMID: 35713318 PMC9828335

[ref19] Lu M. Zhang X. Li W. Li X. Li S. Yin X.. (2024). The effects of CYP2B6 inactivators on the metabolism of ciprofol. PLoS One 19:e0307995. doi: 10.1371/journal.pone.0307995, PMID: 39074104 PMC11285948

[ref20] Luscher B. Maguire J. L. Rudolph U. Sibille E. (2023). GABAA receptors as targets for treating affective and cognitive symptoms of depression. Trends Pharmacol. Sci. 44, 586–600. doi: 10.1016/j.tips.2023.06.009 37543478 PMC10511219

[ref21] Mihara T. Kikuchi T. Kamiya Y. Koga M. Uchimoto K. Kurahashi K.. (2012). Day or night administration of ketamine and pentobarbital differentially affect circadian rhythms of pineal melatonin secretion and locomotor activity in rats. Anesth. Analg. 115, 805–813. doi: 10.1213/ANE.0b013e3182632bcb 22886841

[ref22] Miller B. R. 3rd. McGee T. D. Jr. Swails J. M. Homeyer N. Gohlke H. Roitberg A. E.. (2012). MMPBSA.Py: an efficient program for end-state free energy calculations. J. Chem. Theory Comput. 8, 3314–3321. doi: 10.1021/ct300418h, PMID: 26605738

[ref23] Mizuno T. Higo S. Kamei N. Mori K. Sakamoto A. Ozawa H. (2022). Effects of general anesthesia on behavioral circadian rhythms and clock-gene expression in the suprachiasmatic nucleus in rats. Histochem. Cell Biol. 158, 149–158. doi: 10.1007/s00418-022-02113-0, PMID: 35614272

[ref24] Ni Y. Yu M. Liu C. (2024). Sleep disturbance and cognition in the elderly: a narrative review. Anesthesiol. Perioperat. Sci. 2:26. doi: 10.1007/s44254-024-00066-2, PMID: 40103751

[ref25] Oda Y. Hamaoka N. Hiroi T. Imaoka S. Hase I. Tanaka K.. (2001). Involvement of human liver cytochrome P4502B6 in the metabolism of propofol. Br. J. Clin. Pharmacol. 51, 281–285. doi: 10.1046/j.1365-2125.2001.00344.x, PMID: 11298076 PMC2015030

[ref26] Panda S. Hogenesch J. B. Kay S. A. (2002). Circadian rhythms from flies to human. Nature 417, 329–335. doi: 10.1038/417329a, PMID: 12015613

[ref27] Qin L. Ren L. Wan S. Liu G. Luo X. Liu Z.. (2017). Design, synthesis, and evaluation of novel 2,6-Disubstituted phenol derivatives as general anesthetics. J. Med. Chem. 60, 3606–3617. doi: 10.1021/acs.jmedchem.7b00254, PMID: 28430430

[ref28] Sato Y. Seo N. Kobahashi E. (2005). The dosing-time dependent effects of intravenous hypnotics in mice. Anesth. Analg. 101, 1706–1708. doi: 10.1213/01.ANE.0000184127.67866.2e, PMID: 16301245

[ref29] Shen J. H. Ye M. Chen Q. Chen Y. Zhao H. L. Khan A.. (2021). Effects of circadian rhythm on Narcotrend index and target-controlled infusion concentration of propofol anesthesia. BMC Anesthesiol. 21:215. doi: 10.1186/s12871-021-01445-z, PMID: 34488646 PMC8419887

[ref30] Song Y. Liu Y. Yuan Y. Jia X. Zhang W. Wang G.. (2021). Effects of general versus subarachnoid anaesthesia on circadian melatonin rhythm and postoperative delirium in elderly patients undergoing hip fracture surgery: a prospective cohort clinical trial. EBioMedicine 70:103490. doi: 10.1016/j.ebiom.2021.103490, PMID: 34280784 PMC8318871

[ref31] Song F. Lv X. Meng J. (2019). Propofol induces postoperative depression and inhibits microglial function in mice. Mediat. Inflamm. 2019, 1–6. doi: 10.1155/2019/7651383 PMC6590526 31281228

[ref32] Sousa da Silva A. W. Vranken W. F. (2012). ACPYPE - AnteChamber PYthon parser interfacE. BMC. Res. Notes 5, 367–375. doi: 10.1186/1756-0500-5-367, PMID: 22824207 PMC3461484

[ref33] Sugano A. Murai H. Horiguchi S. Yoshimoto Y. Amano Y. Kimura T.. (2021). Influence of light-dark cycle on delayed recovery from isoflurane anesthesia induced by hypnotics in mice. J. Pharmacol. Sci. 145, 335–339. doi: 10.1016/j.jphs.2021.02.003 33712285

[ref34] Tang X. Li J. Yang B. Lei C. Dong H. (2023). Efficacy of sleep interventions on postoperative delirium: a systematic review and meta-analysis of randomized controlled trials. Anesthesiol. Perioperative Sci. 1:29. doi: 10.1007/s44254-023-00027-1

[ref35] Valdés-Tresanco M. S. Valdés-Tresanco M. E. Valiente P. A. Moreno E. (2021). gmx_MMPBSA: a new tool to perform end-state free energy calculations with GROMACS. J. Chem. Theory Comput. 17, 6281–6291. doi: 10.1021/acs.jctc.1c00645, PMID: 34586825

[ref36] Viitala P. Posti K. Lindfors A. Pelkonen O. Raunio H. (2001). cAMP mediated upregulation of CYP2A5 in mouse hepatocytes. Biochem. Biophys. Res. Commun. 280, 761–767. doi: 10.1006/bbrc.2000.4195, PMID: 11162586

[ref37] Waldvogel KBRLMF. Distribution of GABAA receptor subunits in the human brain. (2021)

[ref38] Wang D. Huang Y. Wang X. Chen X. Li J. Zhang S.. (2020). Circadian differences in emergence from volatile anaesthesia in mice: involvement of the locus coeruleus noradrenergic system. Br. J. Anaesth. 125, 548–559. doi: 10.1016/j.bja.2020.07.012, PMID: 32807382

[ref39] Wang J. Wang W. Kollman P. A. Case D. A. (2006). Automatic atom type and bond type perception in molecular mechanical calculations. J. Mol. Graph. Model. 25, 247–260. doi: 10.1016/j.jmgm.2005.12.005, PMID: 16458552

[ref40] Wang J. Wolf R. M. Caldwell J. W. Kollman P. A. Case D. A. (2004). Development and testing of a general amber force field. J. Comput. Chem. 25, 1157–1174. doi: 10.1002/jcc.20035, PMID: 15116359

[ref41] Weir C. J. Mitchell S. J. Lambert J. J. (2017). Role of GABAA receptor subtypes in the behavioural effects of intravenous general anaesthetics. Br. J. Anaesth. 119, i167–i175. doi: 10.1093/bja/aex369, PMID: 29161398

[ref42] Xu H. Chen M. Yu F. Zhang T. Wu B. (2020). Circadian clock component rev-erbαRegulates diurnal rhythm of UDP-glucuronosyltransferase 1a9 and drug Glucuronidation in mice. Drug Metab. Dispos. 48, 681–689. doi: 10.1124/dmd.120.000030, PMID: 32527940

[ref43] Yin X. L. Li J. C. Xue R. Li S. Zhang Y. Dong H. J.. (2022). Melatonin pretreatment prevents propofol-induced sleep disturbance by modulating circadian rhythm in rats. Exp. Neurol. 354:114086. doi: 10.1016/j.expneurol.2022.114086 35460759

[ref44] Yu L. Liu X. Zhao X. Shan X. Bischof E. Lu H. H. (2024). Ciprofol versus propofol for anesthesia induction in cardiac surgery: a randomized double-blind controlled clinical trial. BMC Anesthesiol. 24, 114727–114748. doi: 10.1186/s12871-024-02795-0, PMID: 39533186 PMC11556191

[ref45] Yu Z. Ran G. Chai J. Zhang E. E. (2024). A nature-inspired HIF stabilizer derived from a highland-adaptation insertion of plateau pika Epas1 protein. Cell Rep. 43:114727. doi: 10.1016/j.celrep.2024.114727 39269902

[ref46] Zhou Y. Dong H. Fan J. Zhu M. Liu L. Wang Y.. (2024). Cytochrome P450 2B6 and UDP-glucuronosyltransferase enzyme-mediated clearance of Ciprofol (HSK3486) in humans: the role of hepatic and extrahepatic metabolism. Drug Metab. Dispos. 52, 106–117. doi: 10.1124/dmd.123.001484, PMID: 38071562

[ref47] Zurek A. A. Yu J. Wang D. S. Haffey S. C. Bridgwater E. M. Penna A.. (2014). Sustained increase in α5GABAA receptor function impairs memory after anesthesia. J. Clin. Invest. 124, 5437–5441. doi: 10.1172/JCI76669, PMID: 25365226 PMC4348961

