# Supplementary figures and images for: A chronopharmacological comparison of ciprofol and propofol: focus on sedation and side effects
Source: Front Mol Neurosci. 2025 Apr 2;18:1567453. doi: 10.3389/fnmol.2025.1567453 (PMC11999936; doi:10.3389/fnmol.2025.1567453)

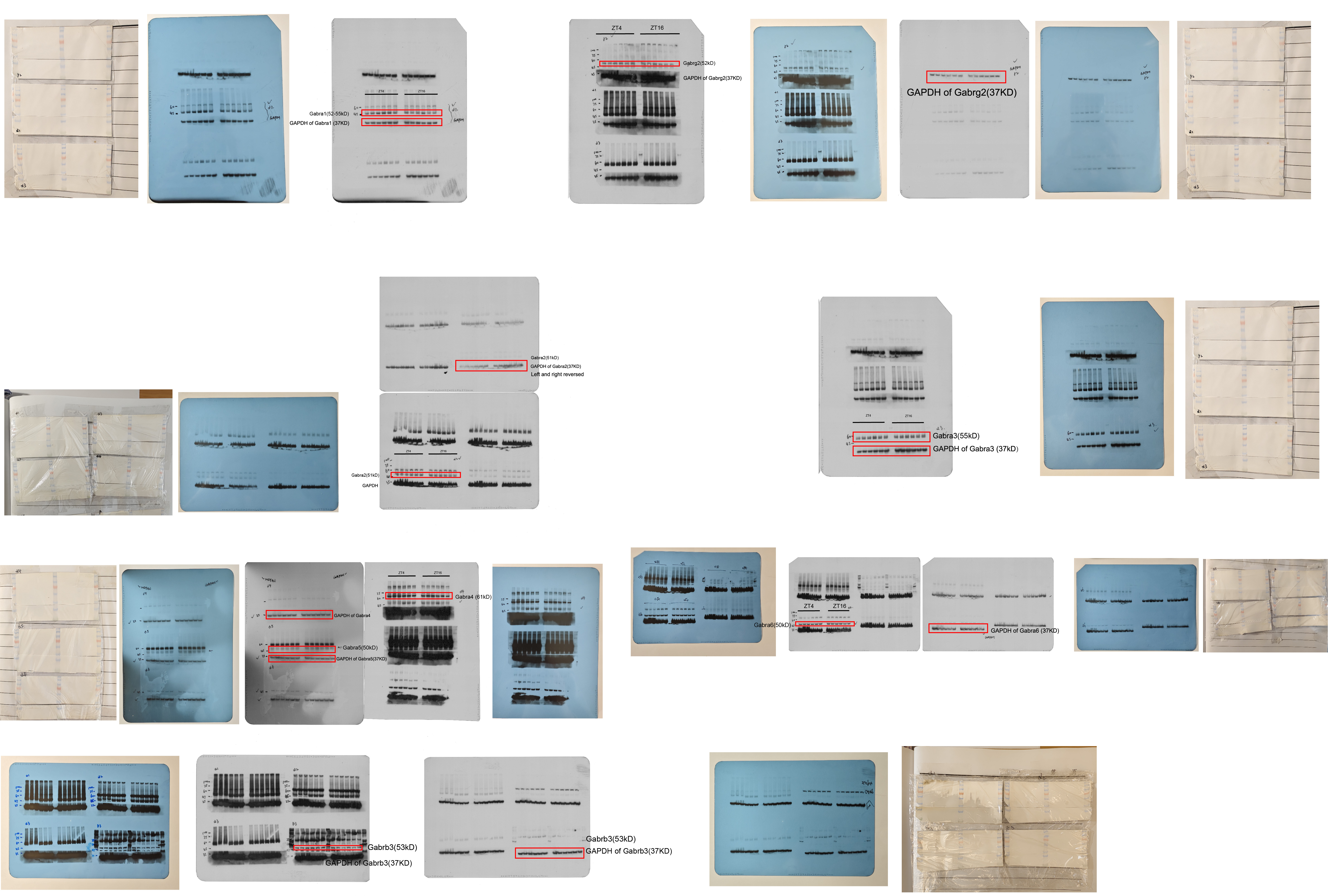

Supplement: Supplementary file 1 [file Image_1.jpeg]

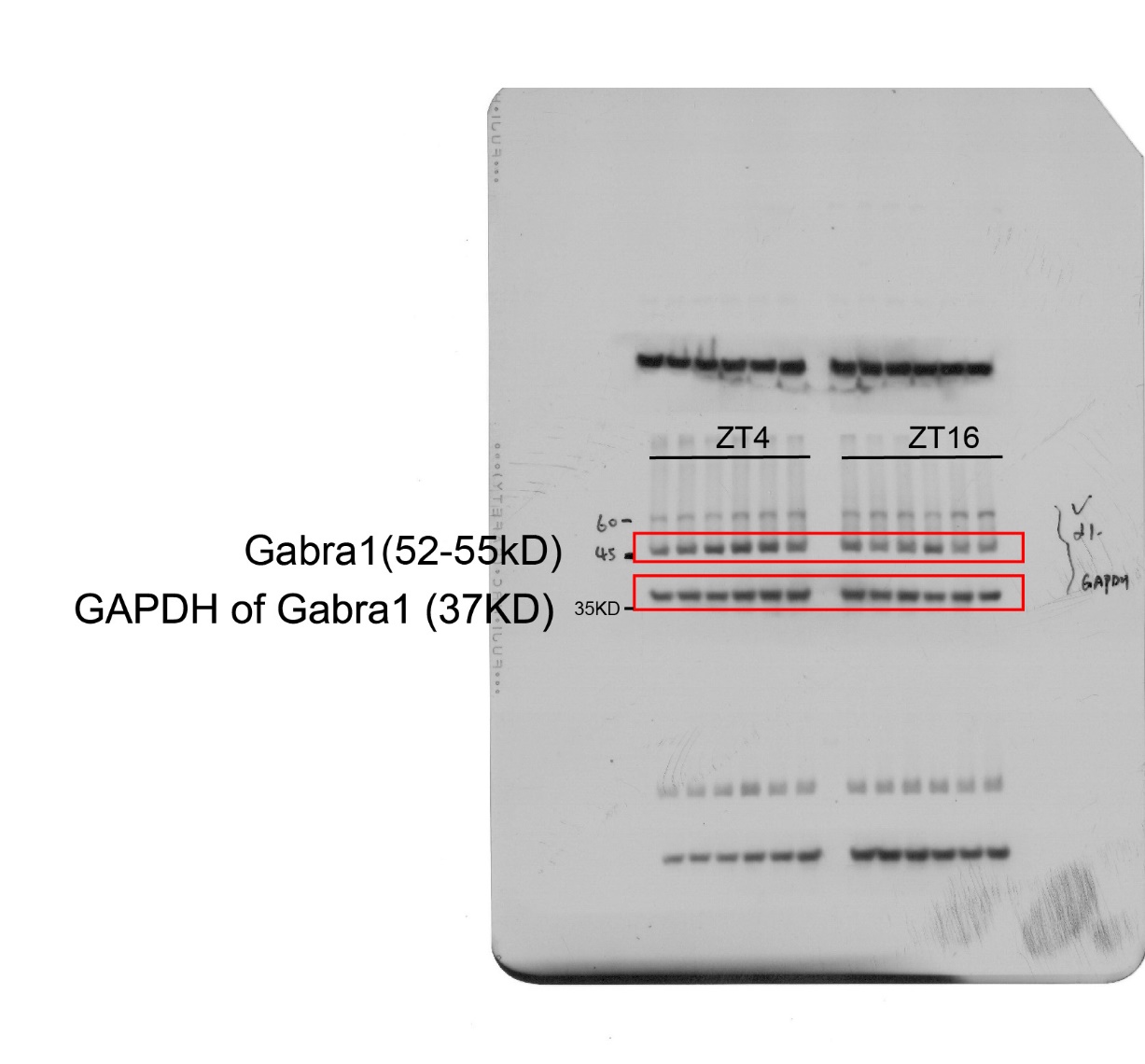


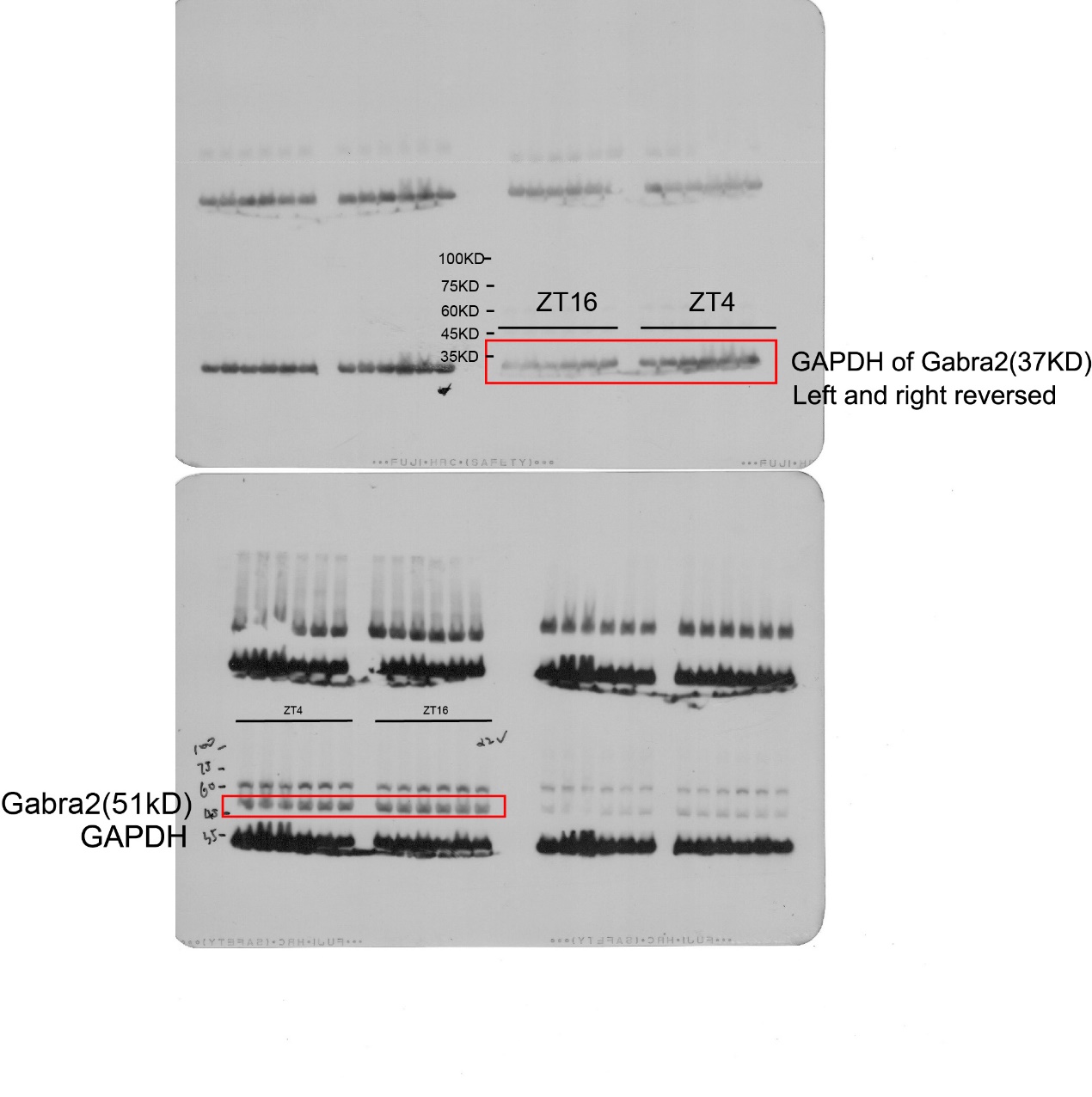

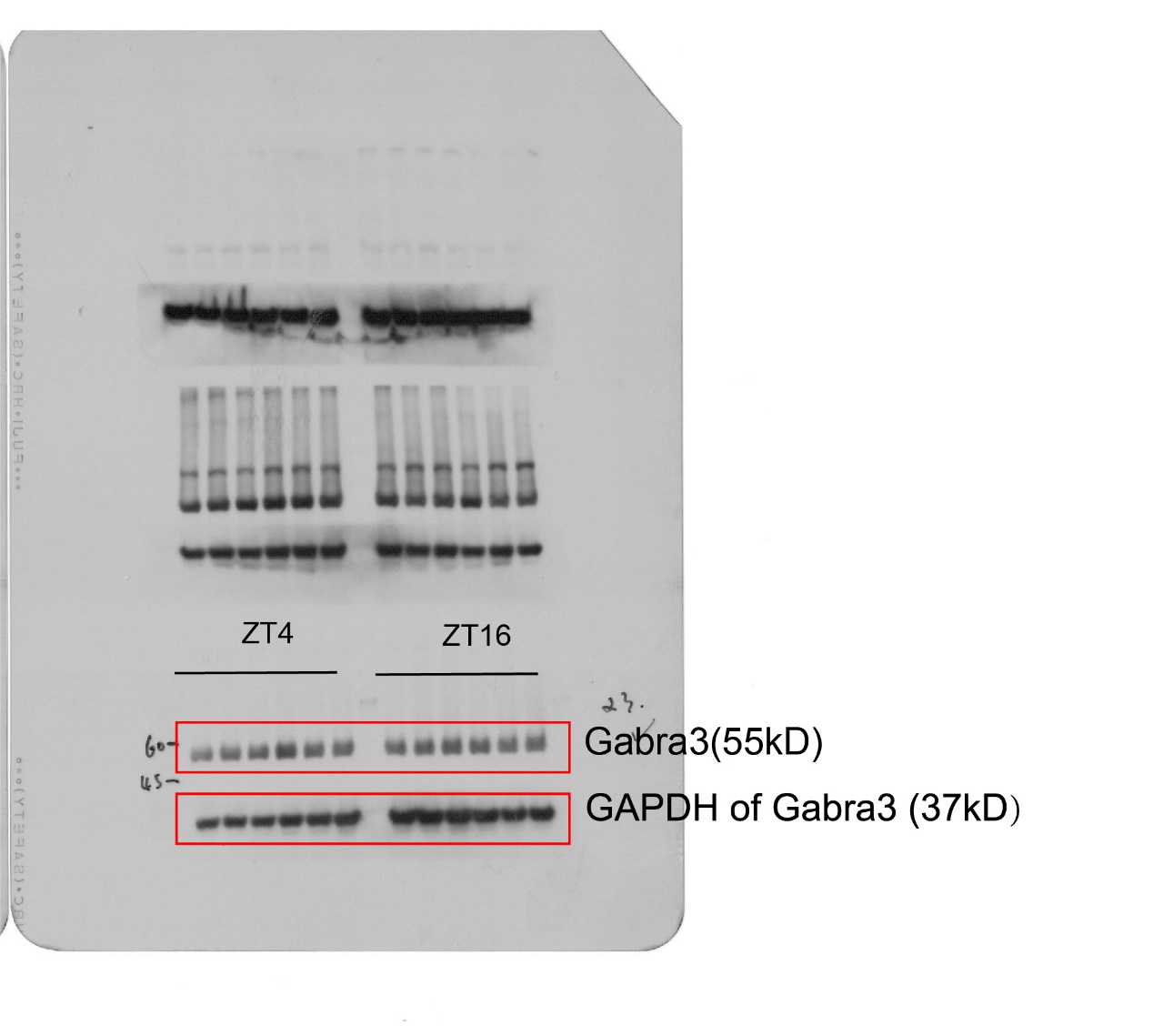


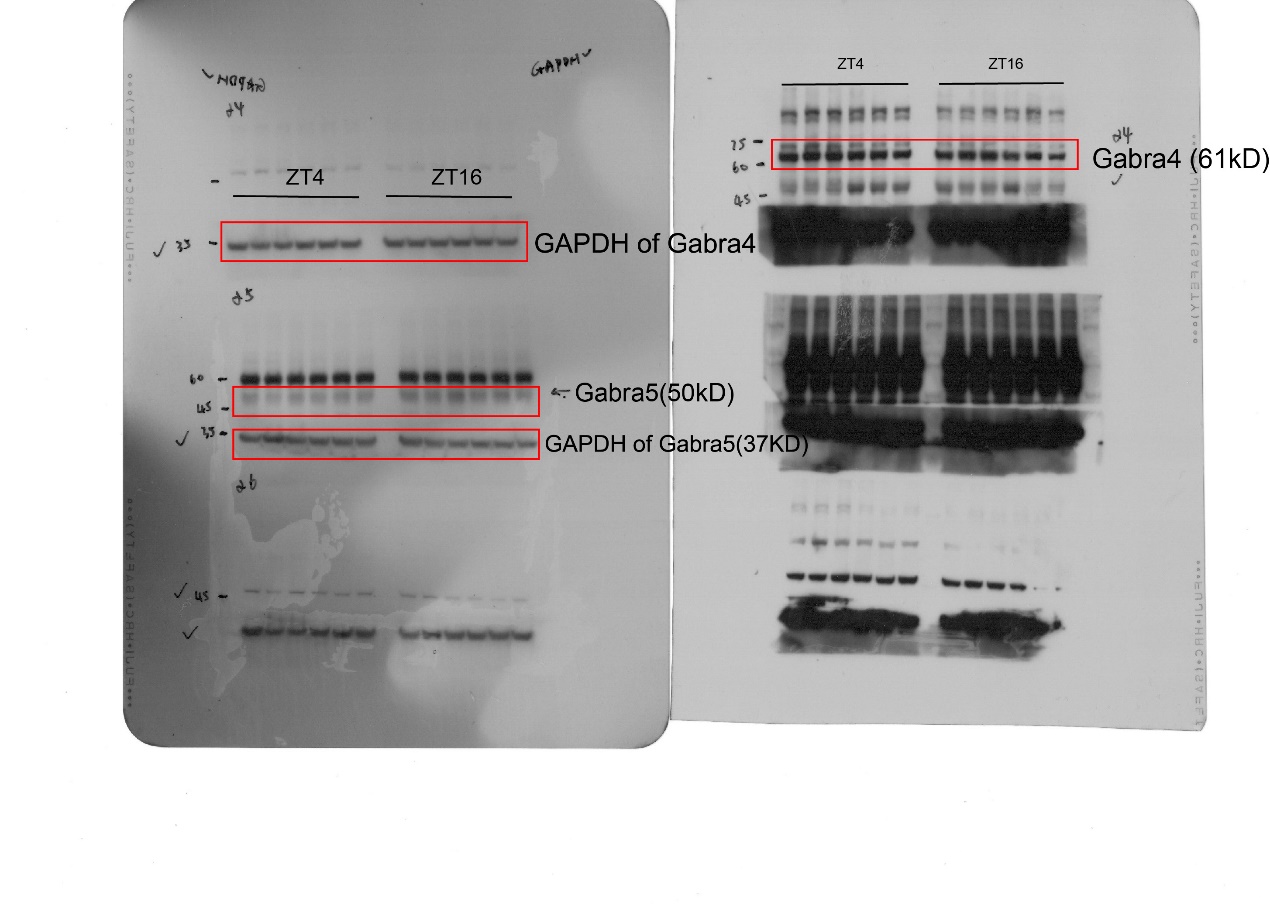


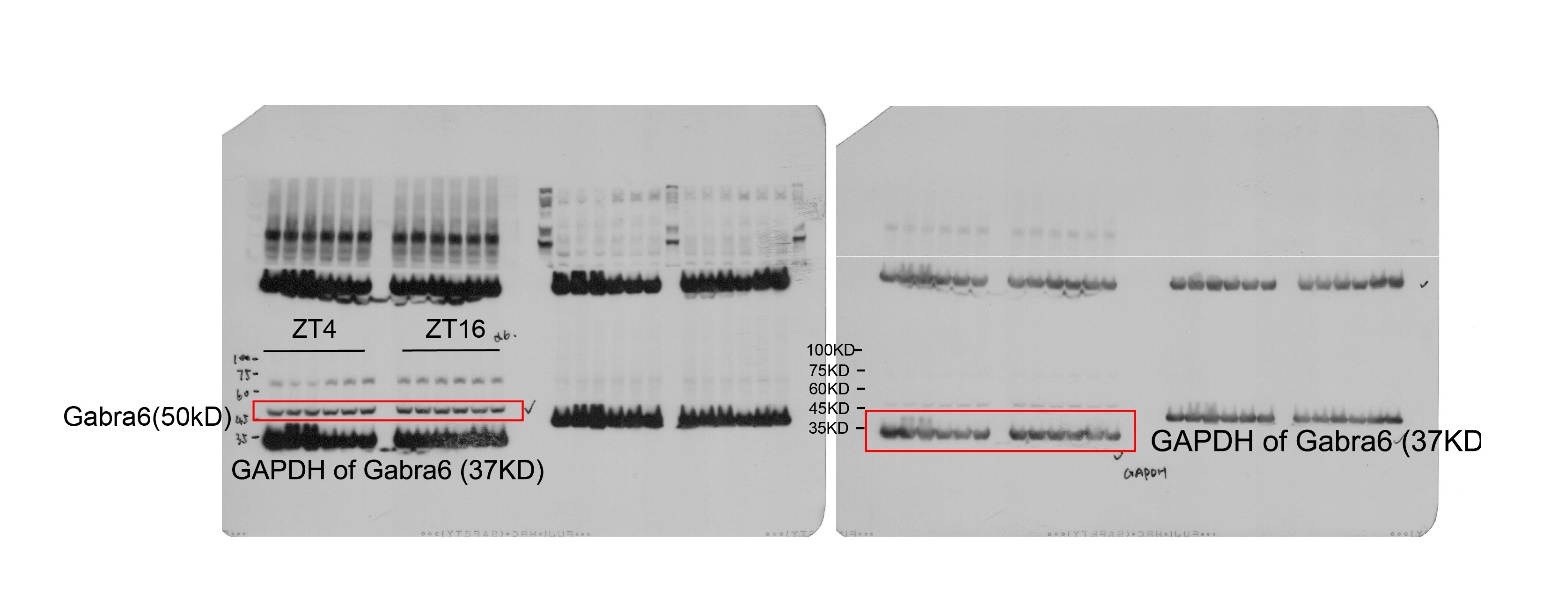


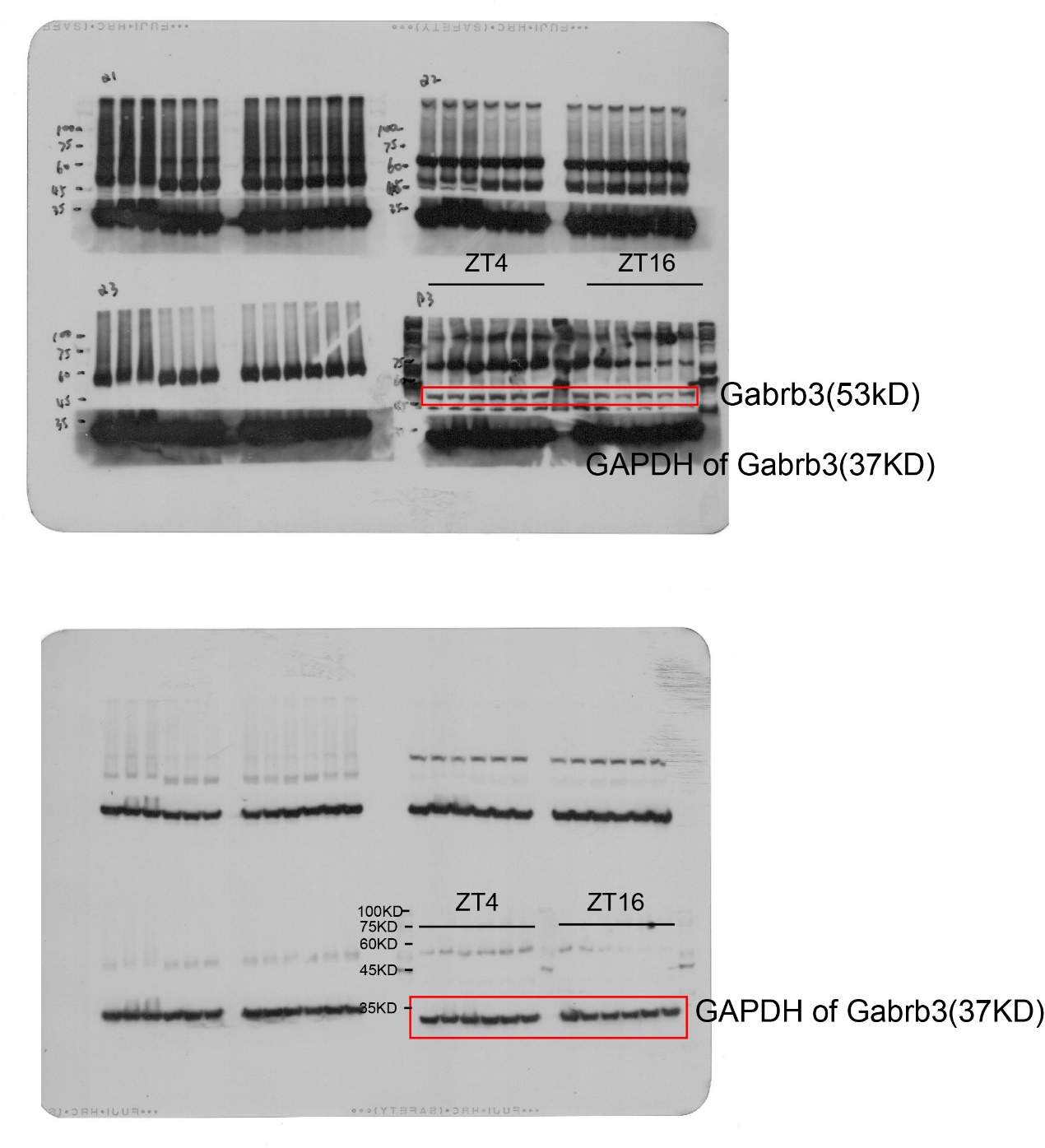


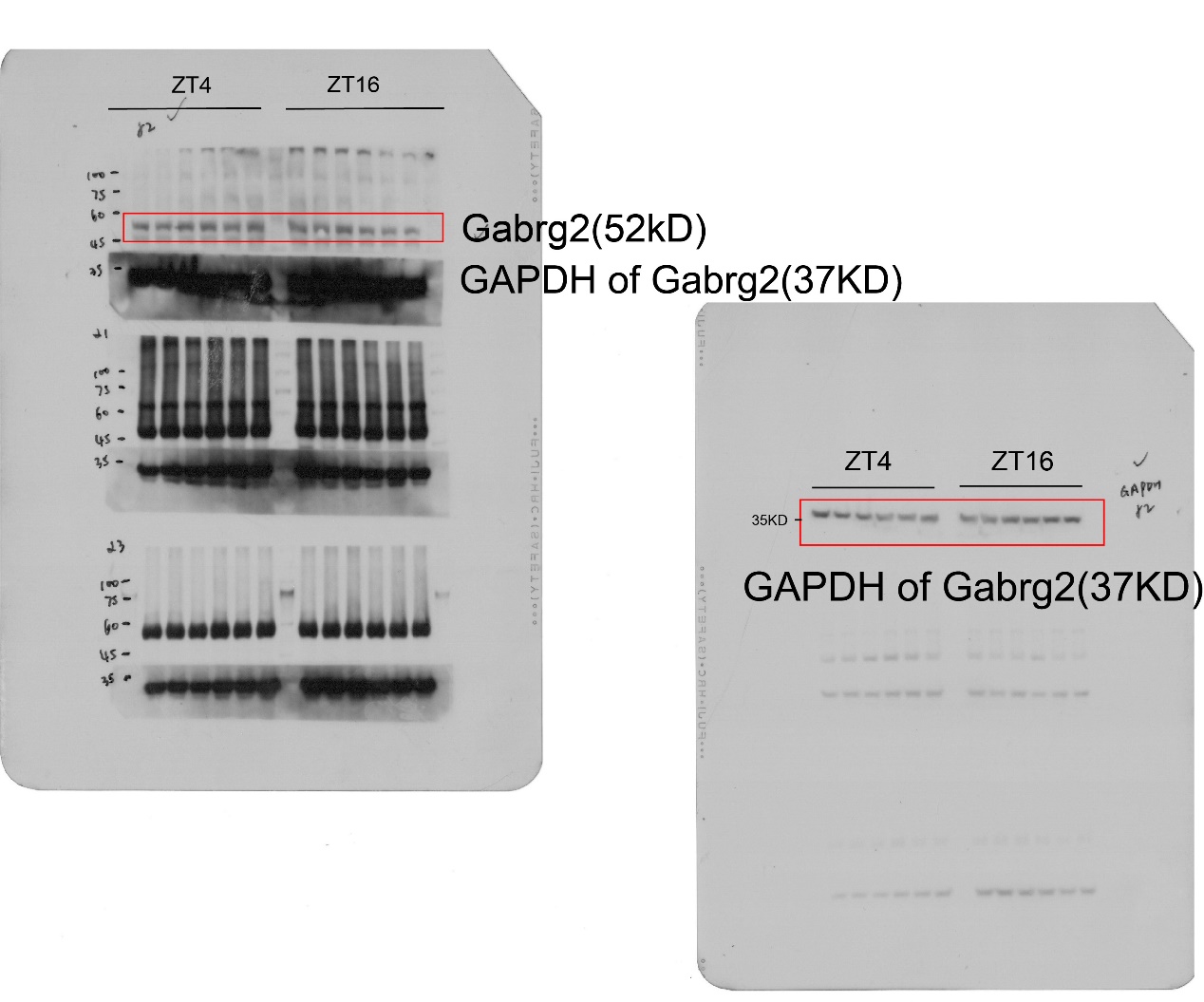

Supplement: Supplementary file 2 [file Table_1.docx]
