# Supplementary material for: A chronopharmacological comparison of ciprofol and propofol: focus on sedation and side effects
Source: Front Mol Neurosci. 2025 Apr 2;18:1567453. doi: 10.3389/fnmol.2025.1567453 (PMC11999936; doi:10.3389/fnmol.2025.1567453)
Supplement: Supplementary file 3 [file Data_Sheet_1.docx]

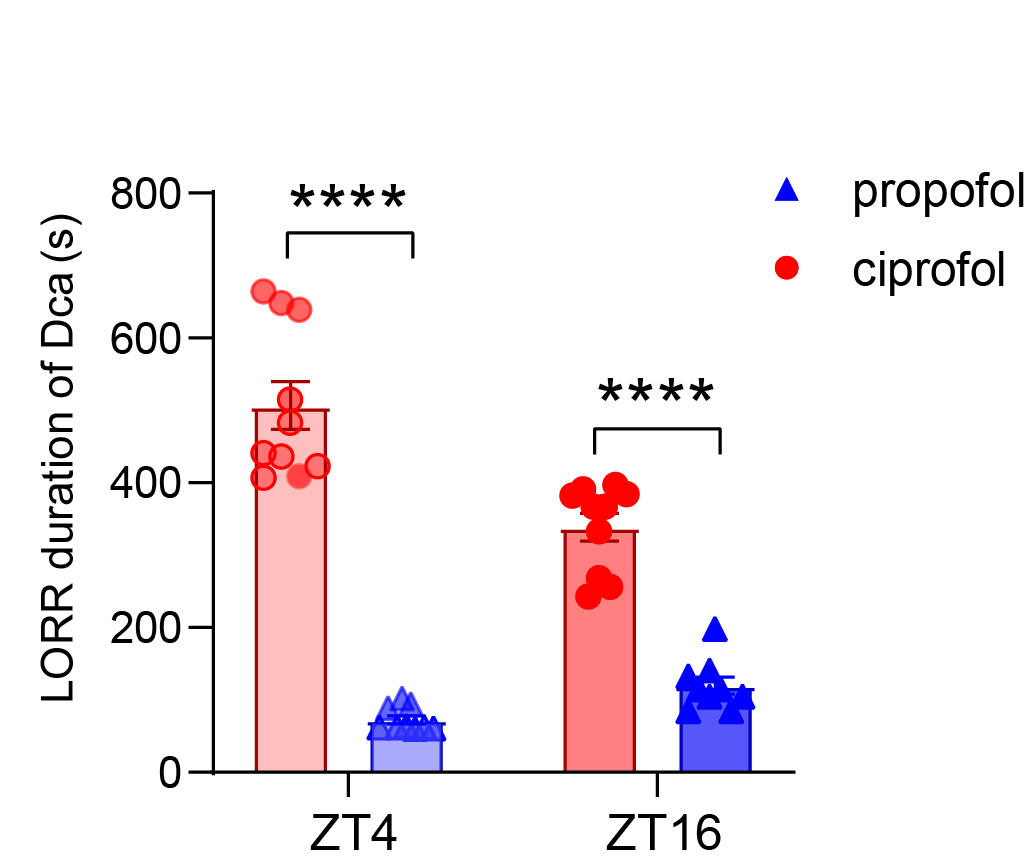


**Figure S1: LORR duration of ciprofol and propofol in ZT4 and ZT16**

LORR duration of ciprofol (5.5 mg/kg) and propofol (11.5 mg/kg) at ZT4 and ZT16 (n = 10 in ciprofol group, n = 9 in propofol group). Statistical analyses were conducted using unpaired unpaired *t*-tests. Data are presented as Means ± SEM. **p* < 0.05, ***p* < 0.01, ****p* < 0.001, *****p* < 0.0001, ns *p* > 0.05.


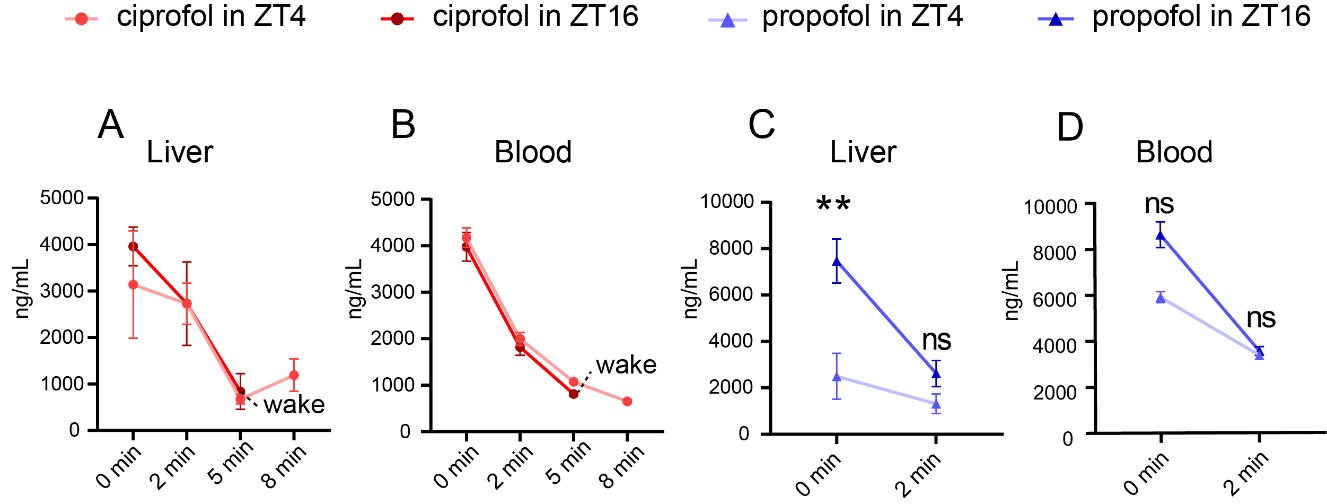


**Figure S2: Distribution of ciprofol and propofol in liver and blood**

**(A-B)** Ciprofol concentration in the liver and blood 0, 2, 5, and 8 min after LORR in ZT4 and ZT16, respectively (n= 3–5). **(C-D)** Propofol concentration in the liver and blood 0 and 2 min after LORR in ZT4 and ZT16, respectively (n= 3 - 5). Statistical analyses were conducted using unpaired *t*-tests. Data are presented as Means ± SEM. **p* < 0.05, ***p* < 0.01, ****p* < 0.001, *****p* < 0.0001, ns *p* > 0.05.


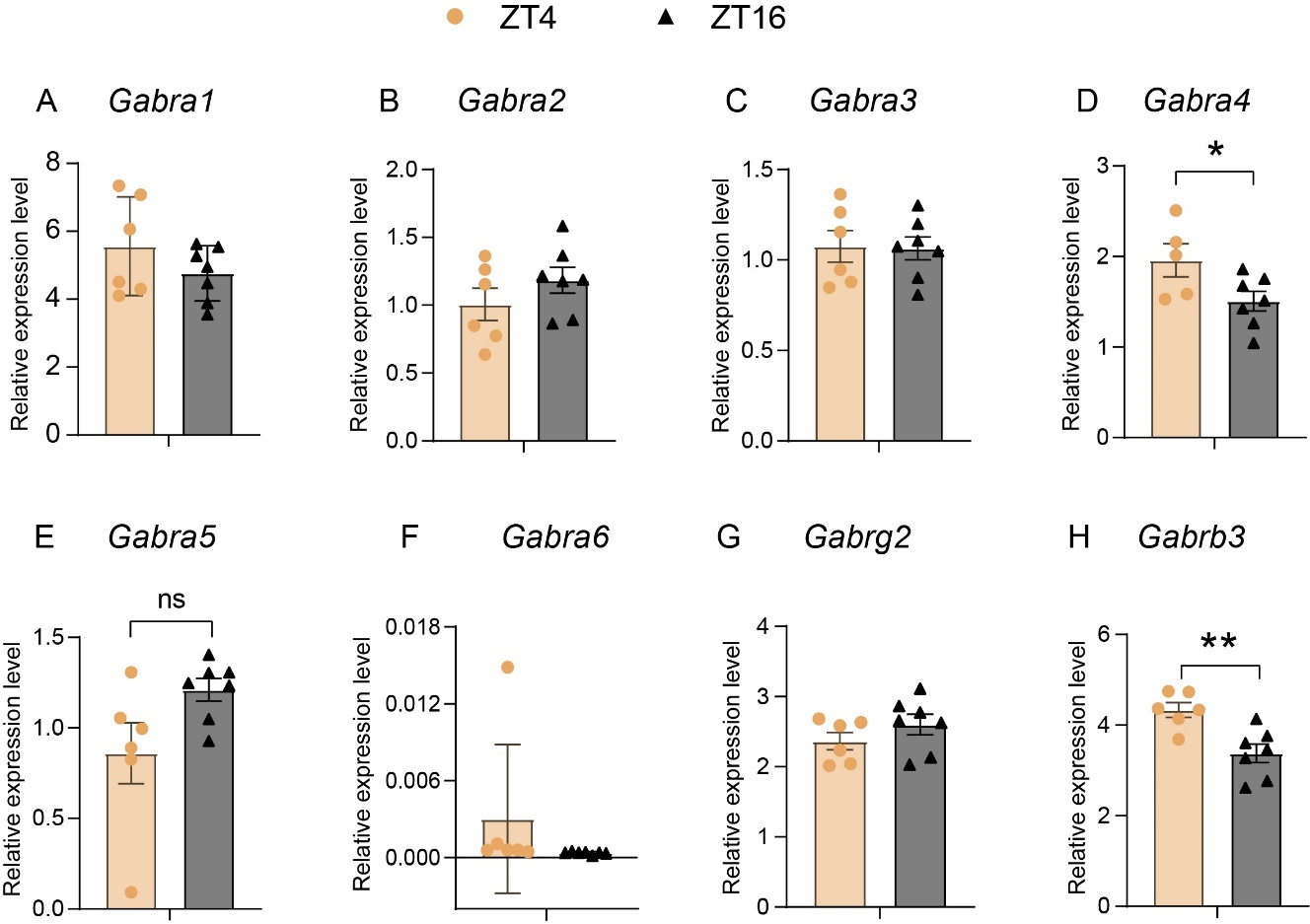


**Figure S3: Expression levels of *Gabra1-6*, *Gabrg2,* and *Gabrb3* at ZT4 and ZT16**

**(A-H)** Expression ratio of *Gabra1-6*, *Gabrg2,* and *Gabrb3* subunits at ZT4 and ZT16 (expression ratio = expression level_GABAAR subunits_ / expression level_GAPDH_) (n = 6). Statistical analyses were conducted using unpaired *t*-tests. Data are presented as Means ± SEM. **p* < 0.05, ***p* < 0.01, ****p* < 0.001, *****p* < 0.0001, ns *p* > 0.05.


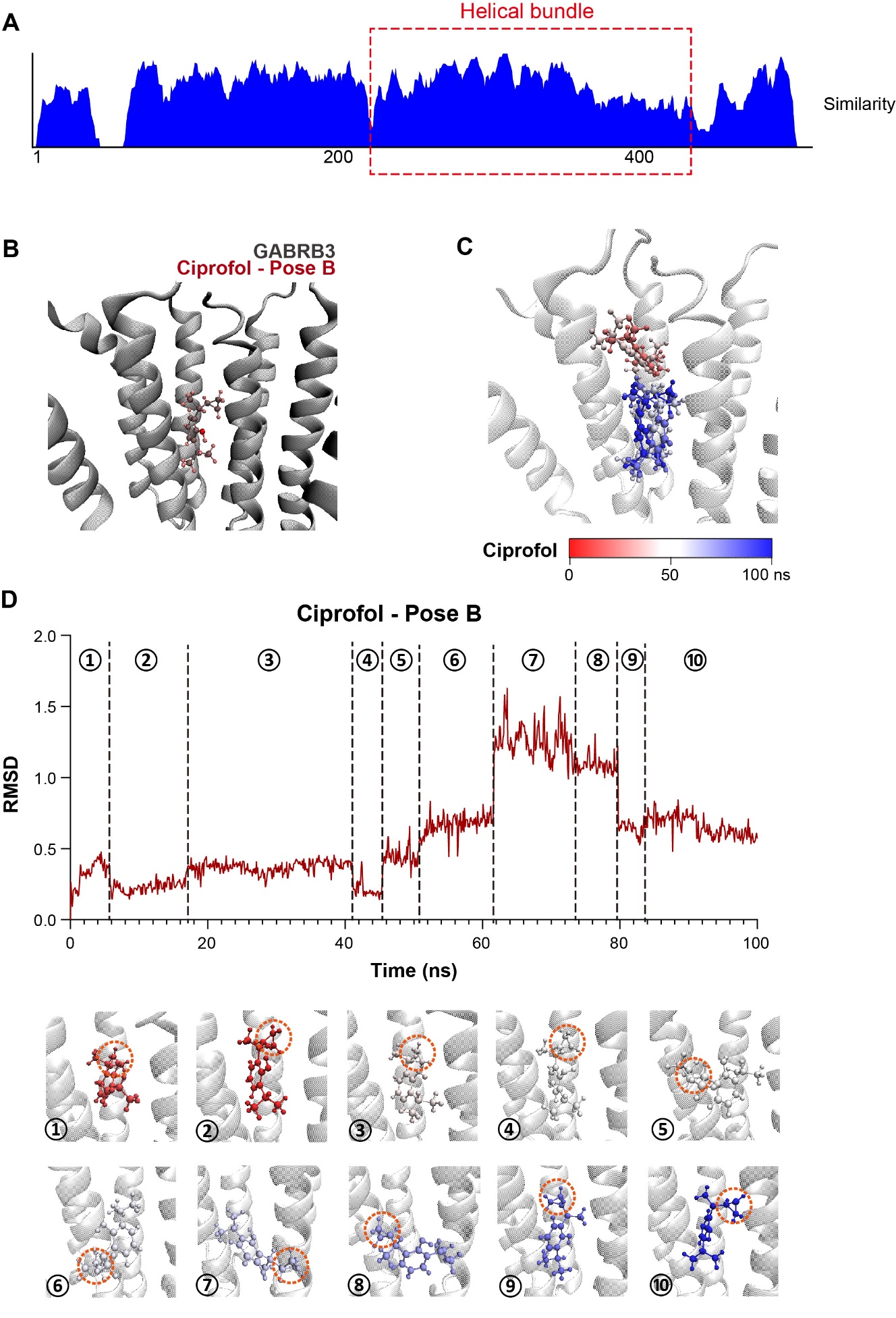


**Figure S4. Ciprofol pose B could even rotate to pose A**

**(A)** Amino acid sequence alignment of different subtypes of GABA receptors. **(B)** Structure model of ciprofol (pose B, dark red) and GABRB3 dimer (gray). **(C)** Conformations of ciprofol (pose B) during molecular dynamics simulation, presented by timestep. (Red to blue, 0 ns to 100 ns.) **(D)** RMSD of ciprofol during simulation and ciprofol conformations at respective stage. (Red to blue, 0 ns to 100 ns.)


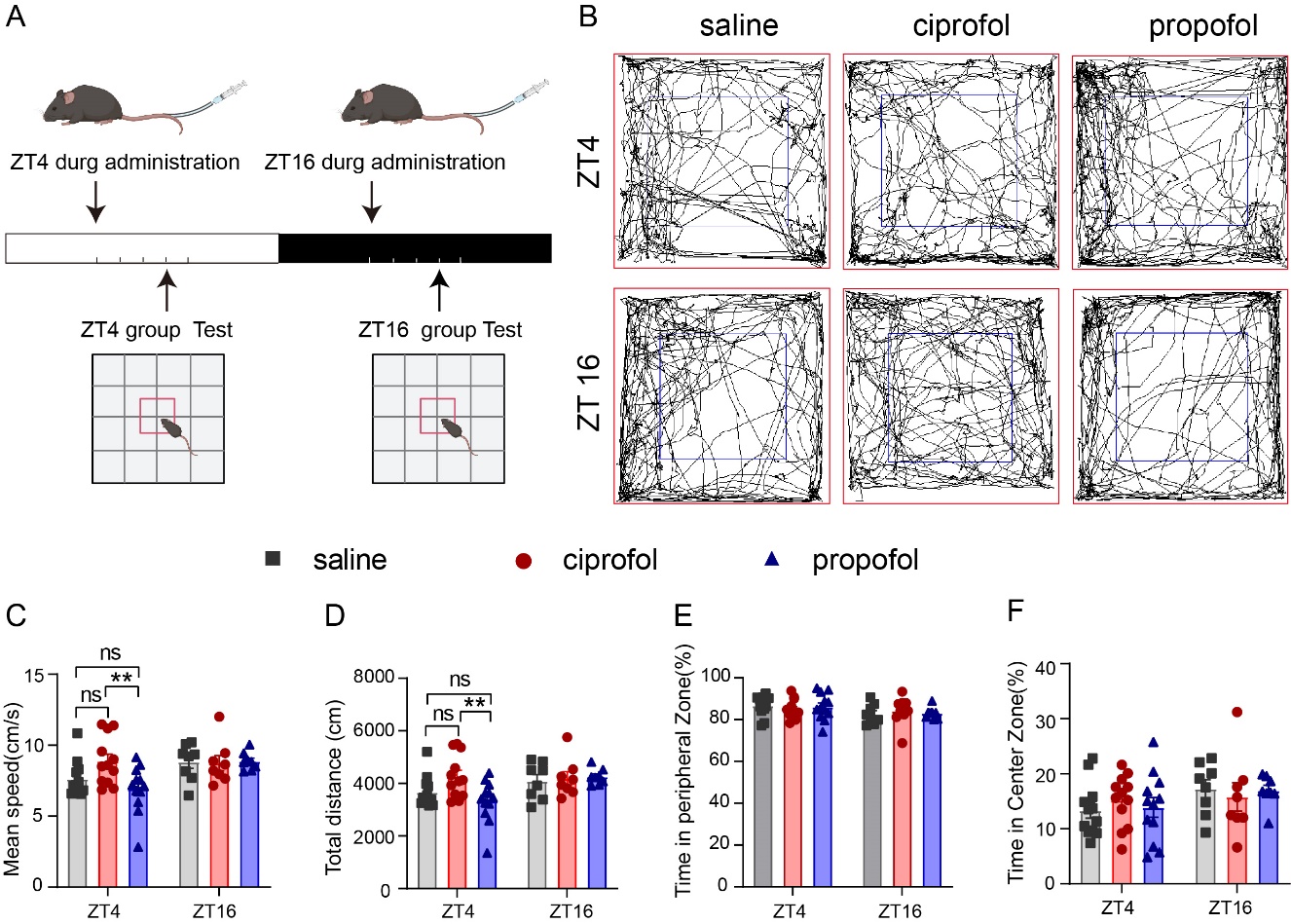


**Figure S5:** **Decreased locomotion induced by propofol compared to ciprofol after 3 h of treatment at ZT4**

**(A)** Experimental scheme diagram. **(B)** Movement and activity trajectories of mice at 3 h after injection of saline (2.75 mL/kg), ciprofol (5.5 mg/kg) and propofol (11.5 mg/kg) in ZT4 and ZT16 (n = 6–12). **(C-F)** Mean speed, total travel distance, time at the peripheral and central zones after the administration of saline (2.75 mL/kg, equal volume of ciprofol), ciprofol (5.5 mg/kg) and propofol (11.5 mg/kg) in ZT4 and ZT16, respectively (n = 6–12).

Statistical analyses were conducted using unpaired *t*-tests. Data are presented as Means ± SEM. **p* < 0.05, ***p* < 0.01, ****p* < 0.001, *****p* < 0.0001, ns *p* > 0.05.


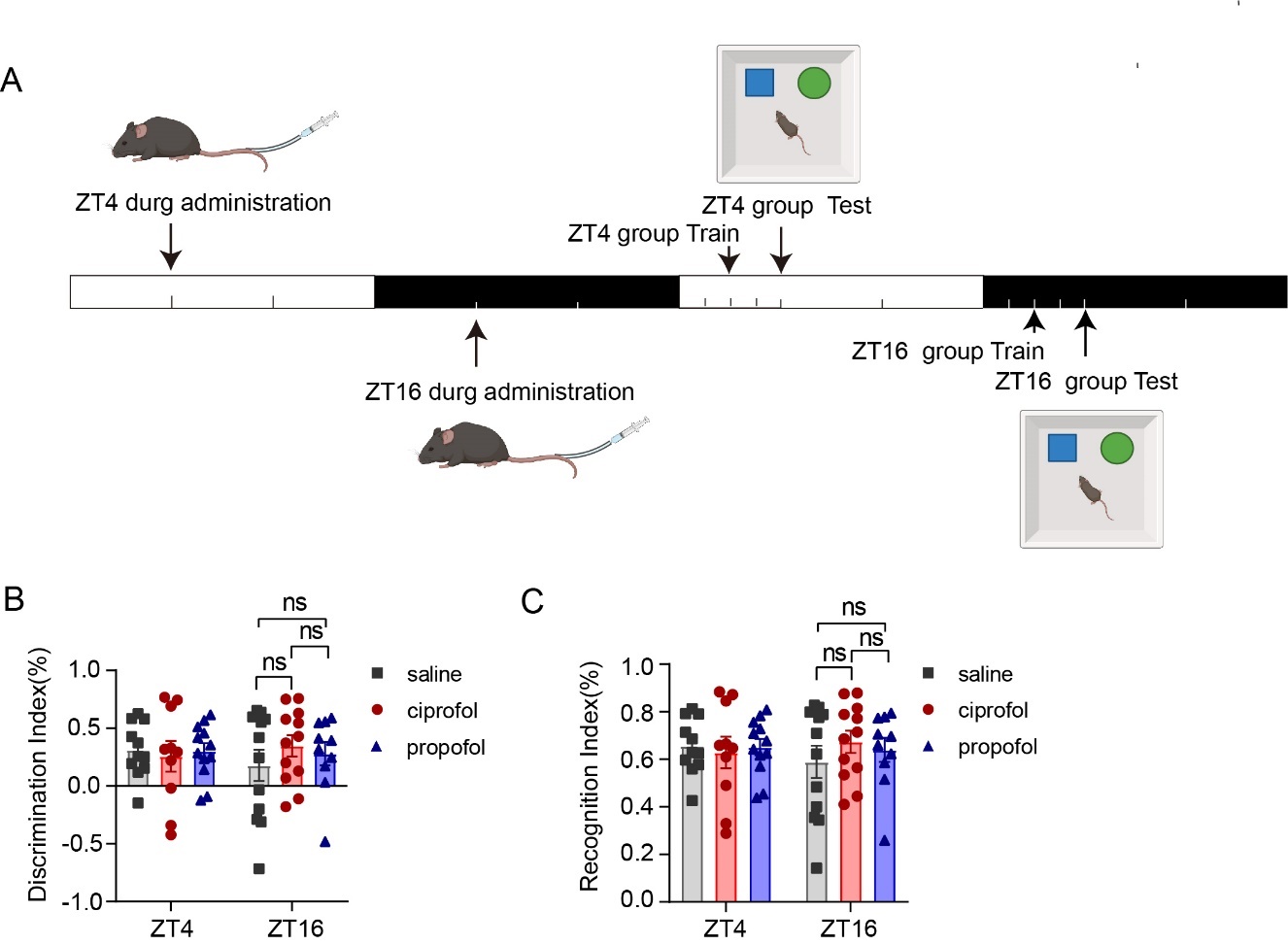


**Figure S6:** **Effects of ciprofol and propofol on cognitive and memory ability after 24 h of ZT4 and ZT16 treatments**

**(A)** Experimental scheme diagram. The familiar period was followed by the test period of the new and old object recognition experiment in 24 h. **(B - C)** Discrimination index (DI) and Recognition index (RI) were measured 24 h after the drug administration in ZT4 and ZT16, respectively (n =10–12). DI = (new - old) / (new + old). RI = new / (new + old). Statistical analyses were conducted using one-way ANOVA with two-stage linear step-up procedure of Benjamini, Krieger and Yekutieli test. Data are presented as Means ± SEM. **p* < 0.05, ***p* < 0.01, ****p* < 0.001, *****p* < 0.0001, ns *p* > 0.05.


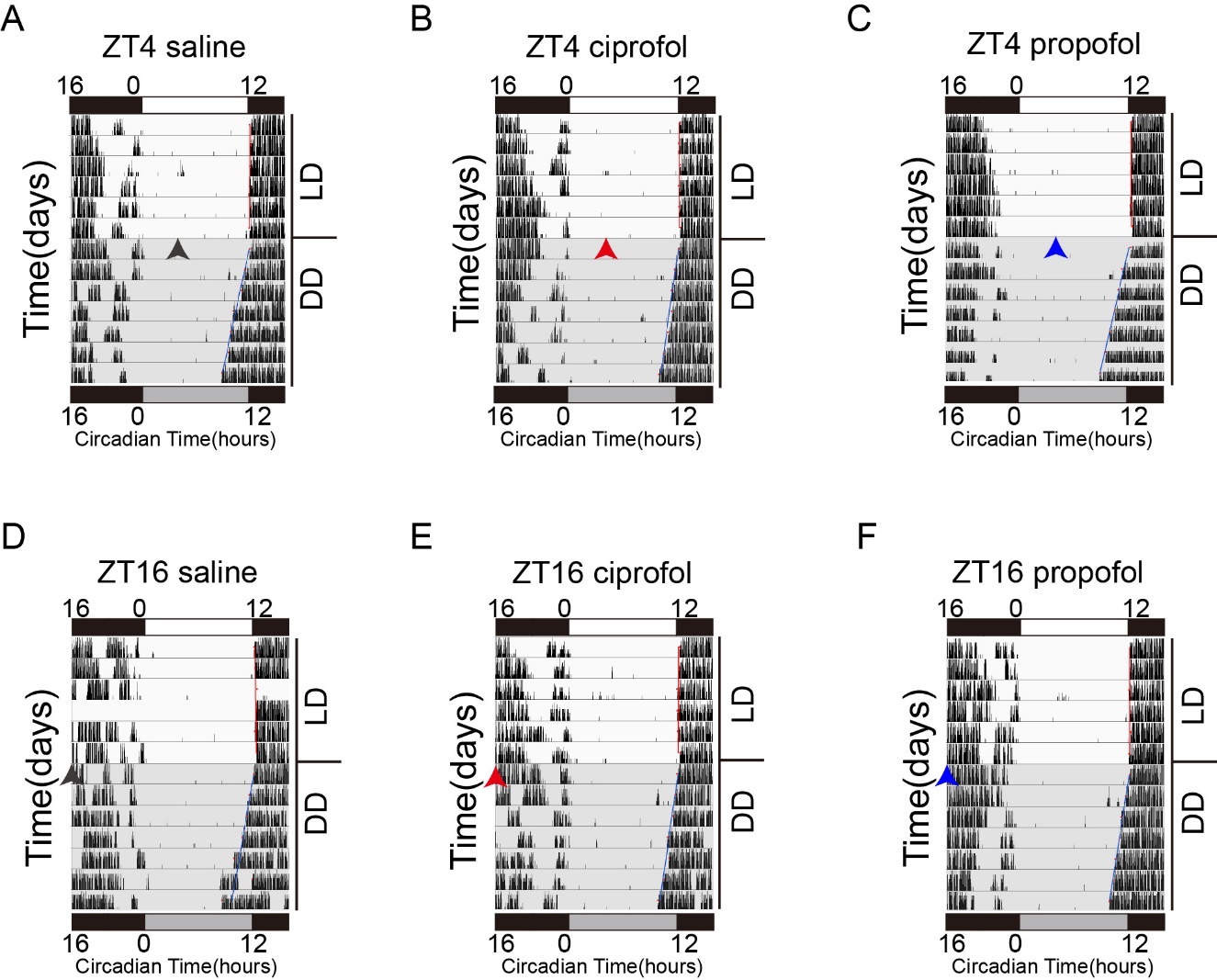


**Figure S7: Effect of ciprofol and propofol on rest/activity rhythm.**
**(A-C)** Drugs were injected at ZT4 after 6 d of LD domestication, and mice were put in DD condition for 1 week. Wheel running was used to record their activity/rest cycle (n = 4–9) for saline (2.75 mL/kg, equal volume of ciprofol), ciprofol (5.5 mg/kg) and propofol (11.5 mg/kg).

**(D-F)** Drugs were injected at ZT16 after 6 d of LD domestication, and mice were put in DD condition for 1 week. Wheel running was used to record their activity/rest cycle (n = 4–9) for saline (2.75 mL/kg, equal volume of ciprofol), ciprofol (5.5 mg/kg) and propofol (11.5 mg/kg).

Activity/rest cycle Data was processed and analysed using ClockLab system (version 6.1.15). Arrow: Drug administration.
